# Endocrine dysfunction in patients with juvenile idiopathic arthritis

**DOI:** 10.1186/s12969-025-01058-7

**Published:** 2025-04-24

**Authors:** Sayan Mukherjee, Abilash Krishnan Vijayakumaran, Mukesh Kumar Maurya, Nishant Gautam Kamble, Ankush PM, Puneet Kumar, Wahid Ali, Mala Kumar, Saurabh Kumar, Pankti Mehta, T. G. Sundaram, Urmila Dhakad

**Affiliations:** 1 Department of Clinical Immunology and Rheumatology, King George’s Medical University, Lucknow, India; 2 Department of Pathology, King George’s Medical University, Lucknow, India; 3 Department of Pediatrics, King George’s Medical University, Lucknow, India; 4 Department of Radiology, King George’s Medical University, Lucknow, India

**Keywords:** Growth dysfunction, Juvenile idiopathic arthritis, Pubertal delays

## Abstract

**Objectives:**

To assess the prevalence of endocrine dysfunction in patients with JIA and identify potential contributory factors for growth and sexual development.

**Methods:**

A prospective observational study was conducted between July 2021 to January 2023, recruited 107 children of JIA fulfilling the revised ILAR classification criteria with disease duration > 6 months, attending Rheumatology department in KGMU, India. Demographic, clinical (anthropometric), and serological (including hormonal) evaluations were assessed at baseline. Growth velocity was recorded after one year. Mann-Whitney U test, chi-square test, and Fisher’s exact t test were applied during statistical analysis.

**Results:**

107 JIA patients were enrolled with a M: F ratio of 2.06:1 (72 boys & 35 girls) with ERA being the most frequent subtype (51.4%). Mean age was 13 (± 4) years with a disease duration of 33 (± 24) months. Mean glucocorticoid intake was 2.17 (± 5.41) mg/day at baseline. 20.6% children were stunted, 22.4% were underweight and 25.2% had low BMI. Stunted children were more likely to have early onset (*p* = 0.015) & high GH level (*p* = 0.013). Underweight children had longer disease (*p* = 0.047) and more damage (*p* = 0.006). Children with weight z < -2 have high GH and low IGFBP3. Low BMI group had high disease activity, damage, and poor quality of life & functional state (p = < 0.01). Delayed puberty was noticed only in 2.8% of children. Girls with low Estradiol level had longer exposure to corticosteroids. Slower growth velocity was observed in 22.4% of children without any identifiable cause.

**Conclusion:**

One third of JIA patients experience growth and pubertal disturbances, primarily due to altered GH-IGF1 axis.

**Supplementary Information:**

The online version contains supplementary material available at 10.1186/s12969-025-01058-7.

## Key messages

• Growth disturbances remain one of the major concerns in management of JIA. Major studies have revealed restricted growth during initial years in specific subtypes of JIA but causative linkage is poorly understood. Lack of studies in Asian countries regarding this evolving issue, where majority is ERA variant.

• Among multiple potential mechanisms, growth hormone resistance and disrupted GH-IGF1 axis is the major driving force. Weight and BMI disturbances precede linear growth, with limited associations of disease activity or corticosteroid therapy. Adolescent girls can have subclinical hormonal dysfunction with longer steroid use.

• This study opens up the alleys for further translational research regarding hormonal dynamics in JIA. In clinical practice, not only pharmacotherapy, but holistic approach to address the concerns of growth and development in young child may improve over-all outcome and well-being of the child with JIA.

**Supplementary Information:**

The online version contains supplementary material available at 10.1186/s12969-025-01058-7.

## Introduction

Juvenile idiopathic arthritis (JIA) is the most common form of childhood rheumatic disease worldwide, with its prevalence in the Indian childhood population being approximately 48/100,000 children [1]. Endocrine dysfunction and growth failure are well-documented complications of a systemic inflammatory disease like JIA [2]. Coexistence of other autoimmune diseases like autoimmune thyroiditis (11.9%), celiac disease (6.6%), and Crohn’s disease (0.07–1.52%) further adds to the problem [3, 4]. Other than systemic inflammation, growth impairment is influenced by various other factors, including long-term use of corticosteroids, undernutrition, changes in body composition with a decrease in lean mass, and limited physical inactivity [5]. Additionally, the degree, extent, and duration of disease activity, as well as the age at disease onset, play significant roles. These factors result in systemic effects on the growth hormone (GH) insulin-like growth factor 1 (IGF-1) axis as well as local effects on the homeostasis and function of the growth plate [6].

The Childhood Arthritis Prospective Study (CAPS) cohort revealed that children with JIA experienced restricted growth during the initial three years of their disease and disrupted linear height velocity over time, and more so in systemic onset JIA and juvenile psoriatic patients [7]. Recognition of growth impairment is important because reduced adult height is a permanent consequence of JIA, affecting 10–20% of these patients [8]. Further studies on pubertal delays reported a prevalence of 15% among JIA, which was significantly higher than healthy controls (1.4%) [9]. Also, it found an association between the dose of GCs, age at GC administration, and delayed puberty in boys. A study from Italy reported isolated delay in timing of menarche in 83 JIA patients compared to their mothers and normal girls [9]. The reason for the causative association wasn’t clear, but it links the dysfunctional hypothalamic-pituitary-gonadal axis to the skeletal maturity.

JIA exhibits a wide spectrum of articular and extra-articular manifestations. Among these, growth failure and delayed puberty pose significant challenges to the physical and psychosocial development of affected children. Despite their profound impact on a growing child’s quality of life, these issues remain underexplored and inadequately addressed in clinical practice.

In the Indian subcontinent, JIA demonstrates unique epidemiological and clinical characteristics, with Enthesitis-Related Arthritis (ERA) being the most common subtype, particularly in Asian populations. The underlying factors contributing to this distinct disease pattern, as well as its effects on growth and pubertal development, remain poorly understood. This gap in knowledge underscores the need for focused research to inform disease control strategies and therapeutic interventions that can support optimal growth and sexual maturation in children with JIA.

This study aims to assess the prevalence of clinical and subclinical endocrine dysfunctions, including abnormalities in growth and puberty, among patients with JIA and identify the potential links between disease activity, therapeutic interventions, and endocrine abnormalities to better understand the underlying mechanisms. By addressing these objectives, the study seeks to advance the understanding of JIA’s impact on growth and development, enabling targeted interventions to enhance the overall quality of life for affected children.

## Materials and methods

This is a single-center prospective observational study conducted between July 2021 to January 2023 in the Department of Clinical Immunology and Rheumatology of a tertiary teaching center in Lucknow, India. The study was approved by the Institutional Ethics Committee prior to study commencement (Ref. Code: **VII-PGTSC-IIA/P17**).

### Patients

This study included all consecutive patients of JIA fulfilling the revised ILAR classification criteria [10] and disease duration of at least six months. It excludes individuals diagnosed with secondary amyloidosis, active macrophage activation syndrome or other genetic growth and sexual developmental disorders.

### Objectives

This study aimed to assess the prevalence of clinical and subclinical endocrine dysfunction in patients with JIA. Another objective was to identify potential contributory factors like disease activity and GC use in growth failure and secondary sexual development.

### Methods

Written informed consent from the parents or guardians and assent from the children (age > 7 years) was taken. No patient or public involvement was encouraged during study design, conduct, reporting, or dissemination of the plan other than their participation with informed consent. Demographic and clinical details regarding age of onset, disease duration, clinical subtype, scholastic performance, vaccination status, comorbidities, and prior drug intake were noted. Drug intake history was stratified according to classes like glucocorticoid (GC), non-steroidal anti-inflammatory drugs (NSAIDs), disease modifying anti-rheumatic drugs (DMARDs) and biologics use. GC use was subdivided into current GC use (yes/no), duration of GC use (long term vs. short term: ≥ 3months vs. < 3 months), and cumulative GC dosage (high ≥ 1100 mg vs. low < 1100 mg). All cutoffs for GC dosage and duration of use were derived from adult studies and aligned with standard treatment guidelines established for other adult rheumatic diseases [11–13]. Baseline NSAID intake was expressed as standard NSAID score and categorized according to high and low values (≥ 50 vs. <50) [14, 15].

A detailed baseline clinical evaluation was done which included clinical manifestation, disease activity parameters, quality of life indices, functional status and articular as well as extra-articular damage index. Disease activity score (Juvenile Arthritis Disease Activity Score 27) [16], damage assessment (Juvenile Arthritis Damage Index) [17], quality of life assessment (Paediatric Rheumatology Quality of Life Questionnaire) [18] and functional indices (Childhood Health Assessment Questionnaire) [19] were noted at baseline as well as at 3rd, 6th, and 12th month follow-up visits.

Anthropometric measurements included height, weight, body mass index (BMI), mid-arm circumference (MAC), arm span, upper segment, and lower segment proportion. Mid-arm circumference was taken only for kids less than 5 years of age, and body mass index (BMI) was calculated for all from their respective height and weight. Our study population doesn’t include neonates. Length is measured for age < 2 years by infantometer and standing height for age > 2 years by stadiometer. Weight is measured by a standard weighing machine. All anthropometric parameters were expressed as absolute numbers as well as age- and gender-matched z scores [20–22]. Growth parameters, including height-for-age, weight-for-age, and BMI-for-age, were individually plotted on standardized growth charts, adjusted for age and sex. World Health Organization (WHO) growth charts were used for children under 5 years, while Indian Academy of Pediatrics (IAP) charts were used for those aged 5–18 years. Values below the 3rd percentile were classified as low, and those above the 97th percentile were considered as high for the respective age. Z-scores for each variable were calculated by subtracting the age- and sex-adjusted mean value from the actual value and dividing the result by the standard deviation. Height was expressed as the height SDS (Standard Deviation Score) to indicate the number of standard deviations from the median.

Parental height was recorded during baseline or subsequent visits, and mid-parental height was calculated to estimate the child’s target height. Mid-parental height SDS was derived similarly by adjusting for age- and sex-specific mean and standard deviation values and was analysed for correlation with the child’s height SDS.

The formulas for calculating mid-parental height are as follows:

- For boys: **Mid-parental height = (Father’s height + Mother’s height + 13) / 2**.
- For girls: **Mid-parental height = (Father’s height + Mother’s height − 13) / 2**.

All height measurements were expressed in centimeters.

Growth velocity at 12 months was calculated by adjusting for their age, and sex and those below the 3rd or above the 97th percentile were classified as slow or fast growers respectively.

Pubertal assessment for both boys and girls was done by Tanner staging, and age-adjusted differences in Tanner stage were documented [23, 24]. Lack of pubic hair growth, delayed puberty, and delayed menarche were documented. The Prader orchidometer was used for assessing testicular volume in males. Delayed puberty was defined as a failure to achieve initial characteristics of sexual maturation by an age that is more than two standard deviations above the mean for the population. In girls, thelarche after 13 or menarche after 16 years, and in boys, testicular volume < 4 ml after 14 years of age were classified as pubertal delay. Pubic hair growth depends on adrenarche also and lesser associations with central pubertal development [25].

Bone ages were estimated by comparing hand radiographs with age- and gender-matched references as per The Greulich & Pyle (GP) Atlas method [26]. Bone age was predicted by comparing the development of age-specific ossification centres of the left hand for those aged above 3 years and knee for those below 3 years of age.

Baseline routine laboratory tests as well as hormonal assays were performed. Serum growth hormone (GH), insulin like growth factor-1 (IGF-1), and IGF-1 binding protein 3 (IGFBP3) levels were measured by the Enzyme-linked immunosorbent assay method and expressed as absolute values as well as age- and gender-matched standard deviation scores (SDS). For evaluation of sexual maturity, serum follicle-stimulating hormone (FSH), luteinizing hormone (LH), estradiol and testosterone levels were measured by chemiluminescence assay. All patients were categorized based on their age- and sex-adjusted hormonal levels (Supplementary material Sect. 1). Patients were followed up at one year and growth velocity was recorded and compared with their respective age-based values.

### Statistical analysis

All numerical values were expressed as mean and standard deviation, while categorical values were expressed in frequency and percentage. The test of association was done by using the Mann-Whitney U test for non-parametric variables and the chi-square test or the Fisher’s exact t test for descriptive variables. Correlation analysis was done by calculating Spearman’s Rho coefficient. Growth parameters, such as height, weight, and BMI, were plotted on WHO growth chart, and IAP growth chart separately considering their age and sex. SPSS v.26 software was used for statistical analysis, and a p value of < 0.05 was deemed statistically significant.

## Results

**Fig. 1:**
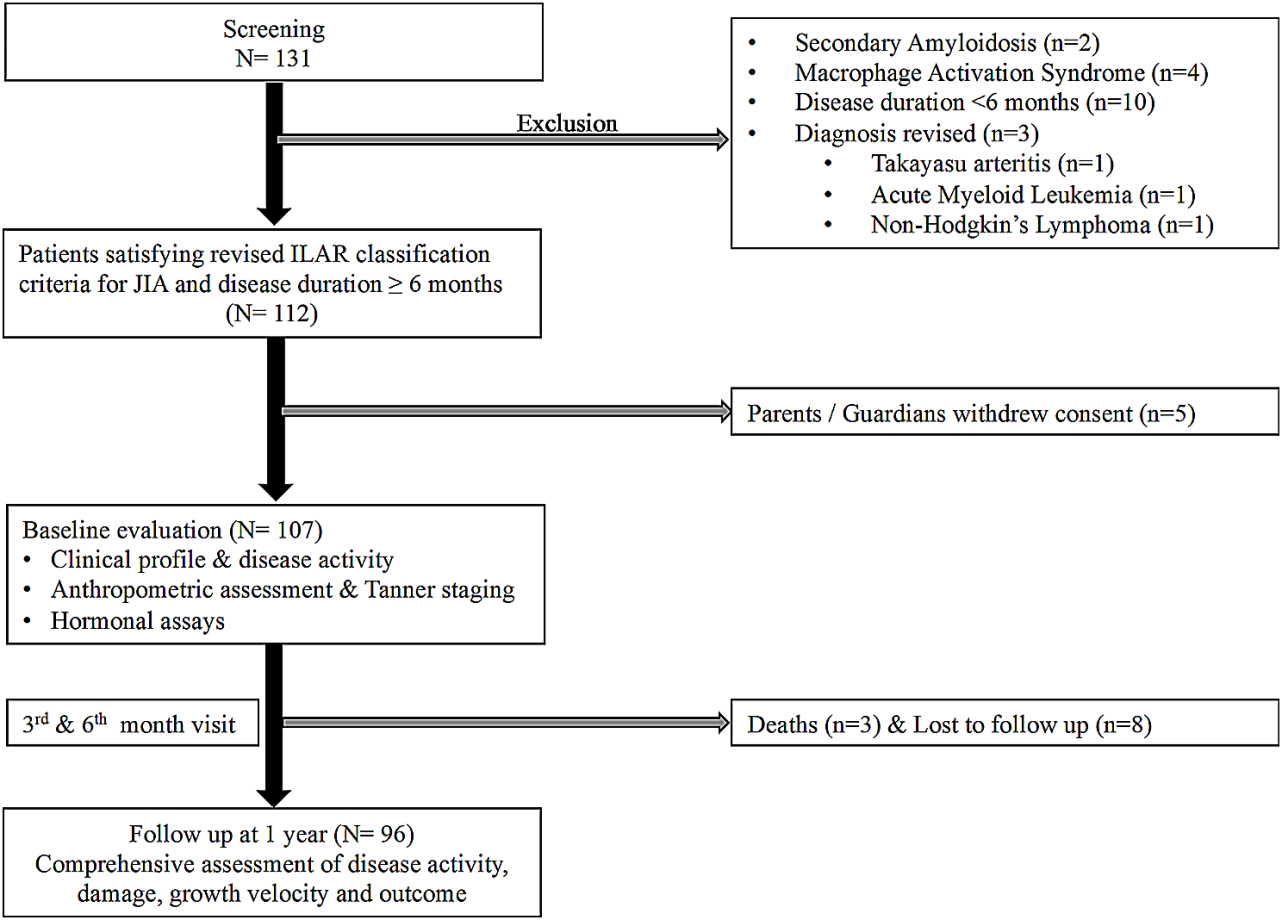
**Study flow diagram**

One hundred and thirty-one children with juvenile idiopathic arthritis were screened, and 107 were enrolled. Among them, 96 patients completed their longitudinal follow up at one year, eight patients resorted to alternative medications and were lost to follow-up, and three patients died before the 1st follow-up visit (Fig. 1).

### Baseline demographic and clinical profile

Out of 107 JIA patients, 72 were boys (67.3%) with a mean age of 13 years (± 4 years). The mean age of onset was 10 years (± 4 years) with a average disease duration of 33 months (± 24 months). Enthesitis-related arthritis (*n* = 55, 51.4%) was the most frequent subtype of JIA among our study population, followed by RF-negative polyarthritis (*n* = 16, 15%). Family history of autoimmunity was observed in 25 (23.4%) children. Among the study participants, enthesitis, plantar fasciitis, and costochondritis were observed in 24 (22.4%), 8 (7.5%) and 4 (3.7%), respectively. Extra-articular manifestations like uveitis, psoriasis and inflammatory bowel disease were seen in 8, 5, and 2 patients, respectively. Joint deformity and contracture were not uncommon (*n* = 25, 23.4%) and were seen across all subtypes of JIA.

Mean daily dose of GC was 2.17 (± 5.41) mg/day (range 0–40 mg/day). The mean duration of GC use was 4.61 (± 10.38) months (range 0–72 months), and the average cumulative dose was 572.17 (± 1260.75) mg (range 0-9250 mg). Steroid use was predominantly restricted to SoJIA and severe Poly JIA, employed as bridge therapy at the discretion of the treating physician. Significant discrepancies between mean and median glucocorticoid usage were noted, driven by higher doses and prolonged use in SoJIA patients without access to IL-1 or IL-6 inhibitors. Adjusted analyses comparing high-dose versus low-dose glucocorticoids were incorporated into the outcome evaluation. Baseline disease activity and functional indices were measured.

### Growth failure in JIA

Anthropometric measurement revealed 22 (20.6%) were stunted, 24 (22.4%) were underweight, and 27 (25.2%) had low BMI for their respective age (Table 1 & Supplementary material Table 2). Stunted individuals were more likely to have an early age of disease onset (*p* = 0.015), a higher growth hormone (GH) level (*p* = 0.013) and be less likely to have enthesitis-related arthritis (*p* = 0.039) in univariate analysis (Table 2). It provides a possible link towards growth hormone resistance in JIA pathogenesis, but multivariate analysis by adjusting traditional, disease, and therapy-related factors failed to show any clear hint (Supplementary material Tables 1 & 6).

**Table 1:** **Baseline growth and pubertal assessment** *(n = 107)*

Parameters		*N* (%)	Median (Range) *[Mean (SD)]*
**Anthropometric measurements**			
Height (*cm*)			147 (78-175.2) *[143.73 (22.48)]*
Height (*For age*)	Short	22 (20.6)	-
	Normal	83 (77.6)	
	Tall	2 (1.9)	
Weight (*kg*)			37 (9-73.4) *[36.04 (14.62)]*
Weight (*For age*)	Normal	83 (77.6)	-
	Low	24 (22.4)	
BMI (*kg/m2*)			16 (10.08-28) *[16.68 (3.68)]*
BMI (*For age*)	Underweight	27 (25.2)	-
	Normal	69 (64.5)	
	Overweight	6 (5.6)	
	Obese	5 (4.7)	
Low Mid arm circumference (*n = 5*)		1 (0.9)	-
Disproportionate arm span		19 (17.8)	-
Disproportionate growth (*US: LS*)		8 (7.5)	-
Limb length defect (*True +/- Apparent*)		11 (10.3)	-
**Radiological assessment**			
Bone age	Delayed	7 (6.5)	-
	Appropriate	96 (89.7)	
	Premature	4 (3.7)	
**Sexual maturity assessment**			
Pubic hair growth			3 (1–5)
Pubic hair growth *(Tanner stage)*	Stage 1	35 (32.7)	-
	Stage 2	10 (9.3)	
	Stage 3	24 (22.4)	
	Stage 4	25 (23.4)	
	Stage 5	13 (12.1)	
Lacks appropriate pubic hair growth		5 (4.7)	-
Breast development *(n = 35)*			2 (1–5)
Breast development *(Tanner stage)*	Stage 1	10 (28.6)	-
	Stage 2	10 (28.6)	
	Stage 3	6 (17.1)	
	Stage 4	7 (20)	
	Stage 5	2 (5.7)	
Male genitalia development *(n = 72)*			3 (1–5)
Male genitalia development *(Tanner stage)*	Stage 1	12 (16.7)	-
	Stage 2	12 (16.7)	
	Stage 3	16 (22.2)	
	Stage 4	13 (18.1)	
	Stage 5	19 (26.4)	
Delayed puberty/ Menarche		3 (2.8)	-

**Table 2:** **Factors affecting growth and development in JIA**

Parameters	Height for age			Weight for age			BMI for age		
	Normal	Short	*p*- value	Underweight	Normal	*p*-value	Normal	Low	*p*- value
	Mean (SD)	Mean (SD)		Mean (SD)	Mean (SD)		Mean (SD)	Mean (SD)	
Age of onset *(years)*	10.6 (3.8)	8.9 (3.0)	0.015*	9(4)	10.6 (3.6)	0.065	10.3(4)	9.9(3)	0.336
Disease duration *(months)*	32 (20)	38 (35)	0.901	44(32)	30(20)	0.047*	30(21)	44(29)	0.012*
Duration of GC use *(months)*	3.8 (8.0)	7.6 (16.6)	0.629	8.1(16)	3.6(7.9)	0.321	3.6(8)	7.7 (15.2)	0.368
Cumulative GC dose *(mg)*	493.3 (937.4)	877.0 (2094.3)	0.686	1014.2 (2056.1)	444.4 (891.7)	0.406	466.8 (966.8)	884.4 (1872.2)	0.432
NSAIDs score	32.22 (28.19)	20.02 (18.07)	0.064	24.41(19.13)	31.24 (28.57)	0.470	29.18 (27.57)	31.28 (24.89)	0.504
PGA	4.4(2.4)	4.2(2.4)	0.846	4.9(2.5)	4.2(2.4)	0.175	4.1(2.4)	4.9(2.3)	0.141
PhGA	4(2)	3(2)	0.389	4(2)	3(2)	0.284	3(2)	4(2)	0.107
JADAS27	12.7 (8.4)	12.1 (8.6)	0.603	14.9(10.6)	11.9 (7.6)	0.378	11.3 (7.4)	16.4 (10.1)	0.029*
JADAS-CRP	12(8)	10.7 (7.6)	0.478	14.1(9.6)	11.1 (7.3)	0.259	10.8 (7.3)	14.6 (9.1)	0.092
JADI-A	2(5)	2(4)	0.273	3(4)	2(5)	0.090	2(4)	4(7)	0.013*
JADI-E	0(1)	1(1)	0.193	1(1)	0(1)	0.006*	0(1)	1(1)	0.001*
PRQoL	9(6)	9(6)	0.988	11(6)	8(5)	0.079	8(5)	11(6)	0.008*
Physical domain	5(4)	5(4)	0.363	6(3)	5(4)	0.084	4(3)	6(4)	0.009*
Mental domain	4(3)	4(2)	0.248	5(3)	4(2)	0.083	4(2)	5(2)	0.019*
CHAQ	0.66 (0.52)	0.53 (0.38)	0.371	0.74(0.52)	0.60 (0.49)	0.238	0.56 (0.45)	0.84 (0.58)	0.023*
Pain-VAS	42(25)	37(25)	0.502	45(26)	40(25)	0.281	39(25)	46(23)	0.223
ESR	43 (33)	46 (35)	0.764	46(37)	43(32)	0.872	38(29)	58(40)	0.029*
CRP	27.3 (28.4)	22.3 (24.8)	0.706	28.6(29.3)	25.6 (27.3)	0.720	24.9 (28.1)	30.1 (26.3)	0.298
GH *(mIU/L)*	7.82 (11.04)	10.71 (10.79)	0.017*	9.31(10.4)	8.15 (11.22)	0.145	8.24 (11.25)	8.93 (10.43)	0.360
IGF-1 *(ng/ml)*	240.70 (142.52)	178.20 (101.15)	0.059	197.21(96.16)	236.71 (145.99)	0.418	234.43 (144.77)	208.33 (110.93)	0.357
IGFBP3 *(ug/ml)*	3.67 (1.58)	3.01 (1.35)	0.061	3.31(1.54)	3.60 (1.56)	0.448	3.58 (1.6)	3.39 (1.44)	0.785

Underweight children exhibited a longer duration of illness (*p* = 0.047) and greater extra-articular damage (*p* = 0.006), underscoring the notion that uncontrolled systemic inflammation in JIA can complicate the disease course (Table 2 and supplementary Tables 3 & 4). In multivariable logistic regression analysis, duration of illness, disease activity score, and ESR showed statistical significant associations with weight-for-age. Furthermore, according to WHO standards, individuals with weight z-scores below − 2 were more likely to exhibit elevated GH levels and reduced IGFBP3 levels (Supplementary material: Table 5). However, in multivariate analysis, z-scores were not significantly associated with any of these variables (Supplementary material: Table 6).

Changes in BMI were more robust, which shows a close association with high disease activity (JADAS27), higher damage (JADI), low quality of life (PRQoL) and lower physical functioning (CHAQ) state of the respective child in the low BMI group (p = < 0.01) (Table 2 and supplementary material Tables 3 & 4). Also, BMI z-score showed a similar relation like higher growth hormone and lower IGFBP3 more frequently in the low BMI for age group (Supplementary material: Table 5). This potentiates the multifactorial nature of growth failure in JIA with a prominent link towards alteration of the GH-IGF1 axis. Even in multivariable logistic regression model, JADAS27 significantly impacted BMI for age and BMI z-scores, which provides a clue that controlling diseases activity by treat to target approach can significantly impact the health and well-being of that child.

### Pubertal dysfunction in JIA

Age-appropriate Tanner stage was attained by 78 (72.4%) children in our study. Delayed puberty and menarche were noticed only in 3 (2.8%) children (Table 1). No statistically significant association was found between age-appropriate Tanner stage attainment and disease- or therapy-related factors (Supplementary material: Tables 8 and 9). Most of the patients had normal gonadotropins with low sex hormones (Supplementary material: Table 7). Low estradiol was seen in 9 (31.4%) girls with a clear association with long-term steroid use (*p* = 0.046) (Table 3). Though 21 (47.2%) boys had low testosterone, but it didn’t reach statistical significance for any disease- or therapy-related factors.

**Table 3:** **Effect of JIA disease activity and glucocorticoid use over sex hormones**

Variables	Categories	Testosterone for age		p-value	Estradiol for age		p-value
		Low *(N = 34)*	Normal *(N = 38)*		Low *(N = 11)*	Normal *(N = 24)*	
		N (%)	N (%)		N (%)	N (%)	
Growth velocity	Normal	22 (71)	25 (73.5)	0.818	8 (88.9)	16 (76.2)	0.426
	Slow	9 (29)	9 (26.5)		1 (11.1)	5 (23.8)	
Disease duration	< 24 months	10 (29.4)	15 (39.5)	0.370	7 (63.6)	6 (25)	0.057
	>=24 months	24 (70.6)	23 (60.5)		4 (36.4)	18 (75)	
Current GC use	Yes	25 (73.5)	25 (65.8)	0.476	8 (72.7)	19 (79.2)	0.674
	No	9 (26.5)	13 (34.2)		3 (27.3)	5 (20.8)	
Duration of GC use	< 3 months	25 (73.5)	27 (71.1)	0.868	5 (45.5)	19 (79.2)	0.046*
	>=3 months	9 (26.5)	11 (28.9)		6 (54.5)	5 (14.3)	
Cumulative GC dose	< 1100 mg	28 (82.4)	31 (81.6)	0.932	8 (72.7)	22 (91.7)	0.137
	>=1100 mg	6 (17.6)	7 (18.4)		3 (27.3)	2 (8.3)	
JADAS27	High + Moderate	31 (85.9)	34 (89.5)	0.807	7 (63.6)	20 (83.3)	0.226
	Low + Inactive	3 (14.1)	4 (10.5)		4 (36.4)	4 (16.7)	

### Linear growth patterns in follow-up

Most of the patients showed normal growth velocity for their respective age during a one year follow-up. We have observed slower growth in 24 (22.4%) children but not attributable to any of those baseline characteristics like disease activity, steroid use, or hormonal levels.

### Hormonal analysis

**Fig. 2:**
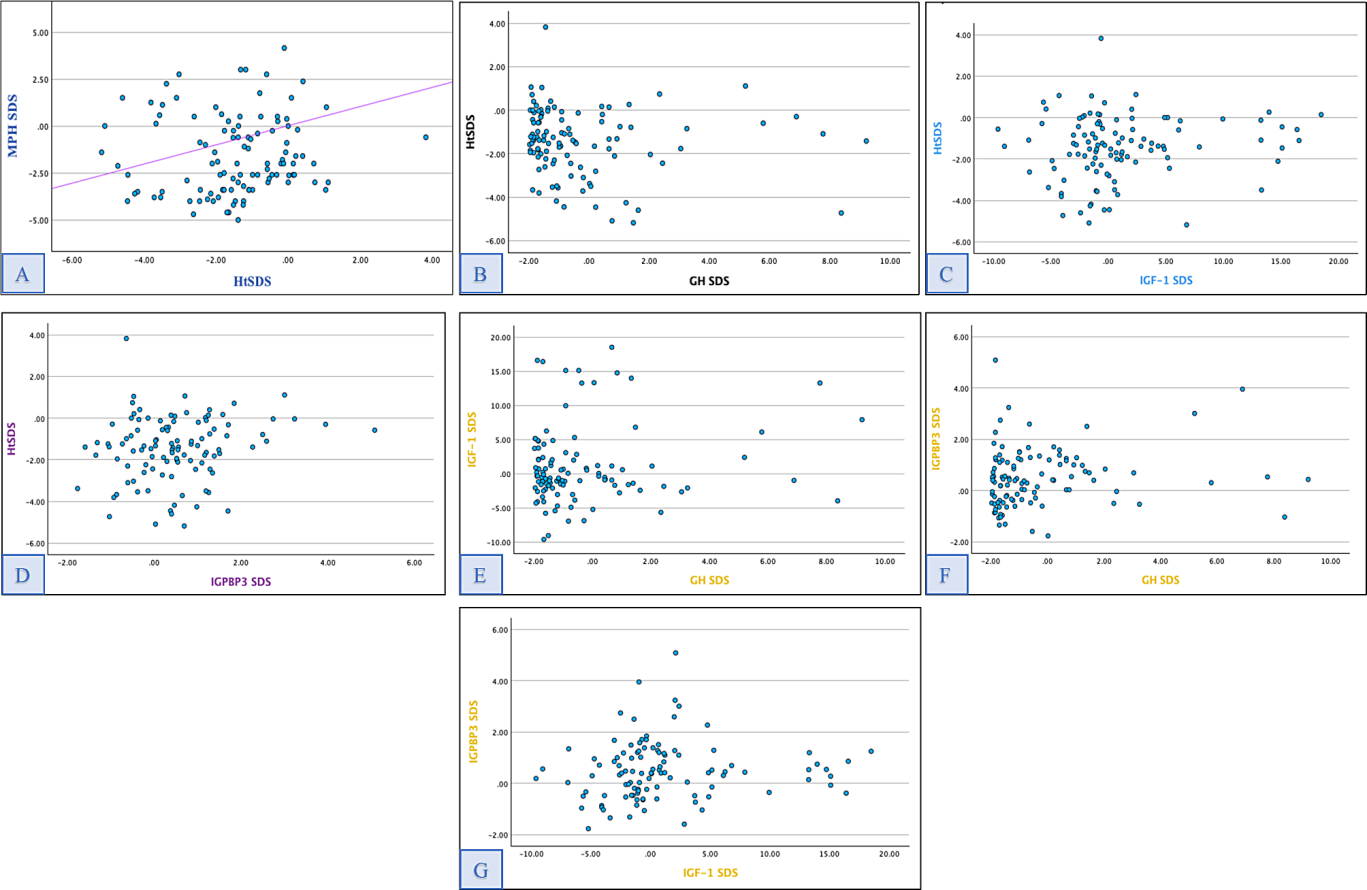
**Age and sex adjusted hormonal scatter plots.** **A**, Height SDS & Mid Parental Height SDS; **B**, Growth hormone SDS & Height SDS; **C**, Height SDS & IGF-1 SDS; **D**, Height SDS & IGFBP3 SDS; **E**, GH SDS & IGF-1 SDS; **F**, GH SDS & IGFBP3 SDS; **G**, IGF-1 SDS and IGFBP3 SDS. *No statistically significant correlation between any of these variables.* (*Diagonal line in figureA represents reference line)*

**Fig. 3:**
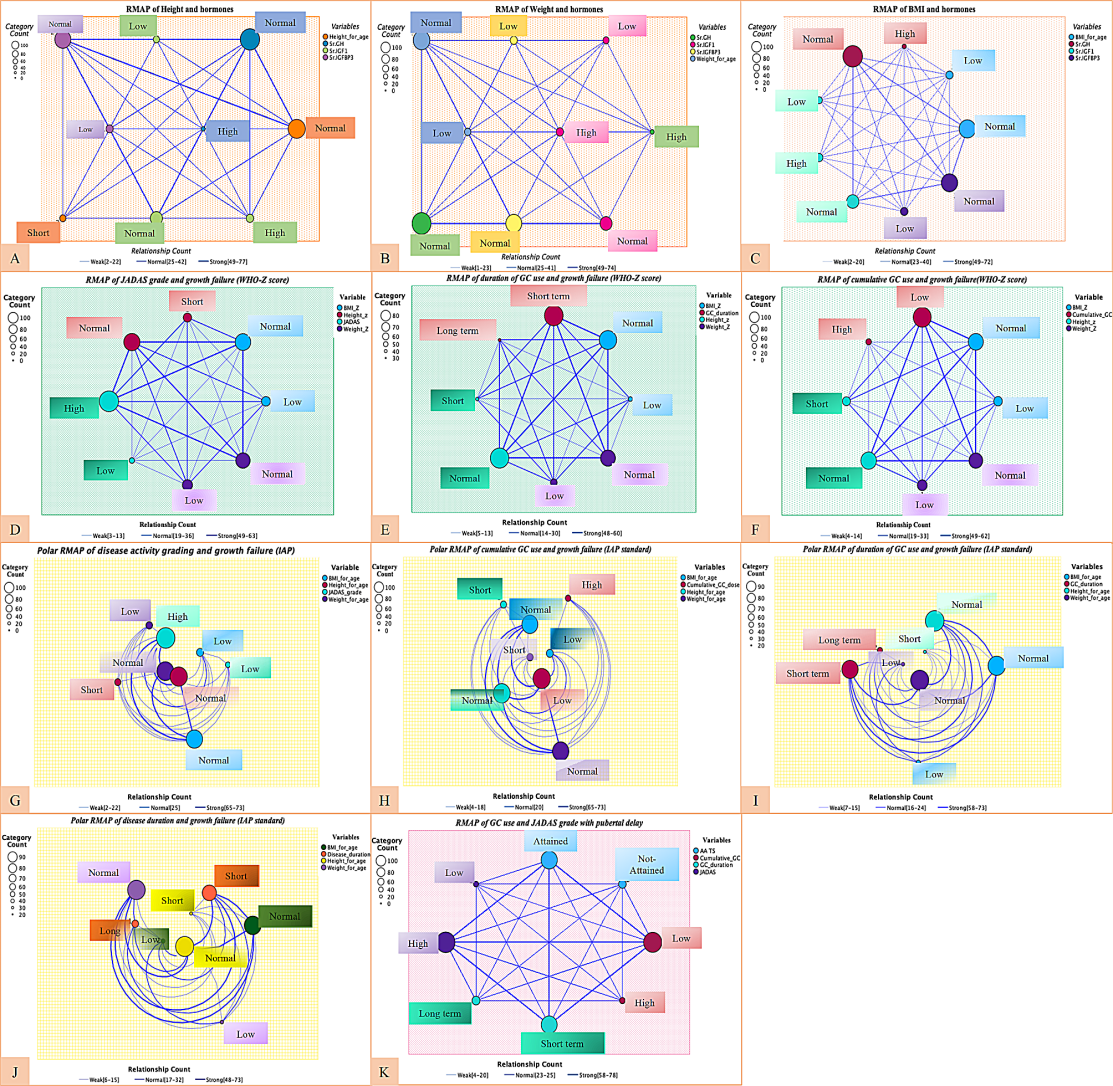
**Relationship Mapping (RMAP) of traditional factors with hormonal levels.** Each square is comprised of a box accompanied with each color coded circles with intervening lines joining those variables. Each circle represents the specific group in that color coded variable (color coding denotes each variable on right) and bottom line represents relationship count which represents the strength of associations between variables (Higher frequency denotes darker line). Size of the circle denotes the number of patients in each circle with a category count expressed on left. (**A**-**C**), Height Weight and BMI relationship with age and sex matched high, low or normal values of GH, IGF-1 and IGFBP3 hormones in a network type relationship map. **D**, disease activity with WHO z scores of height, weight and BMI (z score>-2 is counted as normal). (**E**-**F**), duration and cumulative dose of GC use with WHO z score categories for growth failure. (**G**-**J**), Polar relationship map for describing frequency of associations of IAP standard defined growth failure (below 3 percentile values of age, sex matched Indian population) with disease activity, duration of GC use, cumulative GC dose and disease duration. **K**, disease activity and steroid use with attainment of age appropriate Tanner stage of puberty in a network type relationship map

Baseline basic laboratory profile and hormonal assessments are summarized (Supplementary material: Table 7). No strong relationship was established between disease- and therapy-related factors with serum GH and IGF-1 levels. Neither growth hormone nor IGF-1 standard deviation score correlates well with height standard deviation score (Supplementary material: Table 10; Fig. 2). Also, no correlation was observed between height and mid-parental target height standard deviation score, which describes the non-hereditary pattern of stunted growth. Although the multivariable regression model indicated that IGFBP3-SDS influenced height z-scores, this relationship is likely reflective of an effect rather than a causative phenomenon (Supplementary material: Table 6). Relationship of disease and therapy-related factors with hormonal levels are summarised in Fig. 3.

### Outcome

At one year follow-up, most of the patients achieved low disease activity (*n* = 71, 74%) with half of them achieving clinically inactive disease (Wallace criteria) state. However, a small percentage (*n* = 10, 10.4%) of the patients had persistent high disease activity or experienced a disease flare at follow-up. It’s worth noting that three deaths occurred during the study tenure, and eight participants were lost to follow-up.

## Discussion

Our study offers a comprehensive analysis of the clinical status of children with JIA in a developing country and effectively explores the magnitude of growth and pubertal disorders within this subpopulation. It considers both linear growth (measured through height and growth velocity) and body mass index (BMI) as essential components of growth disorders and looked for potential factors that can predict or contribute to growth disturbances in JIA. By considering both clinical and hormonal aspects, the study contributes significantly to our current understanding of the underlying pathogenic mechanisms and provides valuable insights into the multifaceted nature of growth and pubertal issues in JIA.

Our study demonstrated that the JIA-ERA variant is the most prevalent subtype, with a higher prevalence among boys, a finding that contrasts significantly with observations reported in Western studies [27, 28]. Though country-specific data may vary as per their geographical location, these findings reflect ethnicity-specific prevalence of ERA in the Asian population as documented by several Southeast Asian studies [29, 30]. This chronic, childhood-onset disease significantly impacted the scholastic performance of a considerable number of patients. Additionally, a family history of organ-specific and systemic autoimmunity was noted in 22.3% of children within our JIA cohort. There was no specific predilection for any certain disease type. Coexistence of autoimmune diseases such as vitiligo, hypothyroidism, and Crohn’s disease was observed in a child with JIA. This finding aligns with previous studies and highlights the geographical variability of certain autoimmune diseases [3, 4]. We have diagnosed two patients with subclinical hyperthyroidism and one with type 1 diabetes mellitus in our cohort. We have noticed prior comorbidities related and unrelated to steroid overuse. Comorbidities ranged from acne and striae to more severe manifestations, such as avascular necrosis and multiple vertebral collapses secondary to long-term steroid use, significantly impacting patients’ quality of life.

Our study focused on growth failure and delayed puberty in patients with JIA. We observed that 20.6% of patients had a height below the 3rd percentile for their age, according to the IAP growth chart. However, when comparing the height z-score with age- and sex-adjusted WHO standards, this percentage increased to 29.8%. The prevalence of growth failure in JIA varies (10–41%) depending on the subtype of the disease and the use of baseline corticosteroids [8]. We did not find any statistically significant difference in growth failure based on disease subtype, contrary to previous studies. Interestingly, we observed significant normal growth in ERA compared to the non-ERA population. This could be attributed to the smaller proportion of SoJIA patients (12.1%) and lower baseline GC use in our study compared to the ReACCh-Out cohort, where children received 1 mg/kg/day tapered over 6 months, primarily due to a higher proportion of SoJIA patients [31]. We found significant statistical differences in the mean age of onset and mean growth hormone levels between the short and normal stature groups. However, we did not observe any clear associations with GC therapy or disease activity indices. Comparable results were observed when the height z-scores were analysed in relation to growth hormone SDS levels. These findings suggest that individuals with short stature in JIA are more likely to have an early age of onset and higher-than-normal growth hormone levels. Height disturbances in JIA are more pronounced in early-onset disease, particularly before epiphyseal closure, although affected individuals may exhibit catch-up growth during puberty, similar to healthy children. High to normal growth hormone levels in stunted individuals may give an insight towards GH resistance in JIA pathogenesis. Our study did not demonstrate a meaningful correlation between height SDS and mid-parental height SDS, suggesting that these children aren’t genetically short. Furthermore, only a very small number of patients exhibited delayed bone age, precluding classification as having constitutional delay. There was no strong correlation between child’s height SDS and hormonal SDS like GH, IGF-1 and IGFBP3 in our population as documented by a Korean study in small-for-age babies [32]. Interestingly, very weak negative correlation was observed between child’s height SDS and GH-SDS which may support the theory of growth hormone resistance in JIA. We had longitudinally followed up for one year and documented their height velocity. Although we have not replaced the absolute values with delta z scores, the proportion of slow growers (24.2%) as per age- and gender-matched standard height velocity chart was quite similar to a large prospective study [7]. Growth velocity neither depends on baseline disease activity, steroid use nor on hormonal levels (GH, IGF1, testosterone and estradiol). In chronic conditions like JIA, recombinant growth hormone therapy, as part of a multifaceted approach, has proven effective in overcoming growth challenges by ensuring adequate therapeutic patient education (TPE) and counseling. Effective and meaningful communication between patients, parents and healthcare worker in a structured manner can ensure adherence to GH therapy [33].

Another component of growth failure is weight and BMI assessment. We found 22.4% with low body weight and 25.2% with low BMI for age based on the IAP growth chart and the numbers were even higher when compared with WHO age- and sex-adjusted population. Malnourished children were more likely to have longer disease duration and higher extra-articular damage at baseline, which might be because of uncontrolled inflammation in JIA. Serum GH level and IGFBP3 levels were significantly associated with weight z score. The study showed that higher GH and lower IGFBP3 levels were more likely to be seen in malnourished children. Direct causative linkage cannot be established but this finding could be because of higher stress response of GH with resistance in peripheral tissues in JIA. This is indicative of a disrupted GH-IGF1 axis as seen in other chronic inflammatory diseases [5]. GH-IGF1 axis imparts systemic effects as well as peripheral stimulation in growth plate. Malnutrition can independently cause alterations in the GH-IGF1 axis, leading to both GH insufficiency and IGF-1 resistance [5]. Results of BMI for age were more robust than the weight for age. Underweight population had statistically significant differences in average JADAS27, JADI-A & E, PRQoL (including physical and mental domain), CHAQ and mean ESR values. It indicates that those who had BMI below 3rd percentile for age will be more likely to have higher disease activity and inflammation (high ESR) as well as poor quality of life (PRQoL) and functional status (CHAQ). BMI z-score categories also simulate similar associations with higher articular and extra-articular damages in low BMI group. This is also reflected in the finding of high GH and low IGFBP3 circulating values in individuals with the low BMI.

No determinants like steroid use, high disease activity or hormonal abnormalities were significantly associated with pubertal failures. Normal levels of gonadotrophins were observed in most of the children (*88.2–94.4%*). Low levels of sex hormones were common in boys (*low testosterone – 47.2%)* and girls (*low Estradiol – 31.4%*). It aligns with the current understanding of pathogenesis of pubertal dysfunctions in JIA and majority of children in our study showed hypergonadotropic hypogonadism (gonadal level dysfunction) rather than central pituitary cause (hypogonadotropic hypogonadism). Gonadal dysfunction in both males and females occur due to inadequate 11ßHSD (hydroxy steroid dehydrogenase) active isoform and glucocorticoid receptor downregulation in Leydig cells in testes and granulosa cells in ovary [34, 35]. There was no significant association between gonadal dysfunction and disease activity or steroid use. Only a statistically significant association was seen with the duration of steroid use and estradiol level, which relates to the feedback inhibition of endogenous hormone by iatrogenic steroid use. Preclinical studies on rat ovary showed mRNA expression of 11ßHSD active isoforms, glucocorticoid and mineralocorticoid receptors in granulosa cells of ovary, which are sensitive to LH surge and can regulate sex hormone secretion [36]. So, girls with GC use > 3months are more likely to have a lower estradiol level than short-term GC users. Targeting disease activity with biologics may effectively control diseases in adolescent girls without compromising ovarian reserve [37].

Patients in our study showed a significant mean change in all disease activity, quality of life, and functional indices at one year. NSAIDs score was significantly reduced at follow-up by ensuring a treat-to-target approach and 45.8% of children achieved clinically inactive disease state at one year.

The limitation of our study was small sample size and short follow-up period. Absolute number of patients with growth failure was low, and this may be why statistical significance was not achieved with other predicted factors. Effects of steroids on growth and gonadal failure were not significant in this cohort because of very few SoJIA patients and lesser mean steroid use at baseline both in terms of duration and cumulative dosage used. Growth hormone is itself an acute-phase reactant, and fluctuations in its levels can occur in response to metabolic stress, malnutrition, and inflammation. The GH provocation test, which is a more sensitive method for diagnosing GH deficiency, was not performed, as patients were recruited from both OPD and IPD settings. There is no standardized criterion for diagnosing constitutional delays; therefore, patients were excluded based on discrepancies in bone age estimation, a method that is not universally accepted. Follow-up period was shorter for assessing height velocity and no follow-up was done to record catch-up growth.

## Conclusion

Growth failure are experienced by one third patients of JIA. Exact etiology is still unknown but GH-IGF1 axis dysfunction may be the potential cause. Weight and BMI disturbances may precede linear growth, with limited associations of disease activity or corticosteroid therapy. Pubertal dysfunction can affect both gender, particularly in girls by downregulating estradiol levels with long term corticosteroid use. Comprehensive assessment and longitudinal monitoring, alongside a treat-to-target approach and nutritional support, may improve outcome and well-being of the child with JIA.

## Electronic supplementary material

Below is the link to the electronic supplementary material.

Supplementary Material 1

Supplementary Material 2

## Data Availability

The dataset used/analysed during the current study are available from the corresponding author on reasonable request.

## Abbreviations

ACR: American College of Rheumatology
ACPA: Anti-cyclic citrullinated peptide antibody
ACTH: Adrenocorticotropin hormone
ALP: Alkaline phosphatase
ANA: Anti-nuclear antibody
BMI: Body Mass Index
CHAQ: Childhood Health Assessment Questionnaire
CRP: C Reactive Protein
DXA: Dual Energy X Ray Absorptiometry
DMARD: Disease Modifying Anti-Rheumatic Drug
ERA: Enthesitis related arthritis
ESR: Erythrocyte Sedimentation Rate
EULAR: European League Against Rheumatism
FSH: Follicle-stimulating hormone
GC: Glucocorticoid
GH: Growth hormone
GnRH: Gonadotrophin releasing hormone
HLA: Human Leukocyte Antigen
HbA1C: Glycosylated hemoglobin A1C
IGF-1: Insulin like growth factor − 1
IGFBP3: IGF binding protein 3
ILAR: International League of Associations for Rheumatology
IAP: Indian Academy of Pediatrics
IBD: Inflammatory Bowel Disease
JADAS: Juvenile Arthritis Disease Activity Score
JCA: Juvenile Chronic Arthritis
JIA: Juvenile Idiopathic Arthritis
JRA: Juvenile Rheumatoid Arthritis
JSpADA: Juvenile Spondyloarthritis Disease Activity
NSAID: Non-Steroidal Anti-Inflammatory Drug
LH: Luteinizing hormone
MPH: Mid parental height
PGA: Patient’s / parent’s global Assessment
PhGA: Physician global Assessment
PRQoL: Pediatric Rheumatology Quality of Life
RF: Rheumatoid Factor
SDS: Standard deviation score
sJADAS: Systemic Juvenile Arthritis Disease Activity Score
SoJIA: Systemic Onset Juvenile Idiopathic Arthritis
VAS: Visual analogue scale
WHO: World Health Organization

## References

[1] Abujam B, Mishra R, Aggarwal A. Prevalence of musculoskeletal complaints and juvenile idiopathic arthritis in children from a developing country: a school-based study. Int J Rheum Dis. 2014;17(3):256–60. 24405528 10.1111/1756-185X.12276

[2] Alhomaidah D, Alsagheir A, Al-Mayouf SM. Coexistence of endocrinopathies in children with rheumatic diseases. Int J Pediatr Adolesc Med. 2016;3(3):119–22. 30805481 10.1016/j.ijpam.2016.04.002 PMC6372428

[3] Stagi S, Giani T, Simonini G, Falcini F. Thyroid function, autoimmune thyroiditis and coeliac disease in juvenile idiopathic arthritis. Rheumatology. 2005;44(4):517–20. 15695302 10.1093/rheumatology/keh531

[4] Simon TA, Harikrishnan GP, Kawabata H, Singhal S, Brunner HI, Lovell DJ. Prevalence of co-existing autoimmune disease in juvenile idiopathic arthritis: a cross-sectional study. Pediatr Rheumatol. 2020;18(1):43. 10.1186/s12969-020-00426-9 PMC7275412 32503658

[5] Wong SC, Dobie R, Altowati MA, Werther GA, Farquharson C, Ahmed SF. Growth and the growth hormone-insulin like growth factor 1 Axis in Children with chronic inflammation: current evidence, gaps in knowledge, and future directions. Endocr Rev. 2016;37(1):62–110. 26720129 10.1210/er.2015-1026

[6] Gaspari S, Marcovecchio ML, Breda L, Chiarelli F. Growth in juvenile idiopathic arthritis: the role of inflammation. Clin Experimental Rheumatology-Incl Supplements. 2011;29(1):104. 21269577

[7] McErlane F, Carrasco R, Kearsley-Fleet L, Baildam EM, Wedderburn LR, Foster HE, et al. Growth patterns in early juvenile idiopathic arthritis: results from the Childhood Arthritis prospective study (CAPS). Semin Arthritis Rheum. 2018;48(1):53–60. 29217290 10.1016/j.semarthrit.2017.11.002 PMC6089842

[8] Simon D, Fernando C, Czernichow P, Prieur AM. Linear growth and final height in patients with systemic juvenile idiopathic arthritis treated with longterm glucocorticoids. J Rhuematol. 2002;29(6):1296–300. 12064849

[9] Rusconi R, Corona F, Grassi A, Carnelli V. Age at menarche in juvenile rheumatoid arthritis. J Pediatr Endocrinol Metabolism: JPEM. 2003;16:285–8. 12729405

[10] Petty RE, Southwood TR, Manners P, Baum J, Glass DN, Goldenberg J, et al. International League of Associations for Rheumatology classification of juvenile idiopathic arthritis: second revision, Edmonton, 2001. J Rhuematol. 2004;31(2):390–2. 14760812

[11] Boers M, Hartman L, Opris-Belinski D, Bos R, Kok MR, Silva JAD, et al. Low dose, add-on prednisolone in patients with rheumatoid arthritis aged 65+: the pragmatic randomised, double-blind placebo-controlled GLORIA trial. Ann Rheum Dis. 2022;81(7):925–36. 35641125 10.1136/annrheumdis-2021-221957 PMC9209692

[12] Smolen JS, Landewé RBM, Bergstra SA, Kerschbaumer A, Sepriano A, Aletaha D, et al. EULAR recommendations for the management of rheumatoid arthritis with synthetic and biological disease-modifying antirheumatic drugs: 2022 update. Ann Rheum Dis. 2023;82(1):3–18. 36357155 10.1136/ard-2022-223356

[13] Ramiro S, Nikiphorou E, Sepriano A, Ortolan A, Webers C, Baraliakos X, et al. ASAS-EULAR recommendations for the management of axial spondyloarthritis: 2022 update. Ann Rheum Dis. 2023;82(1):19–34. 36270658 10.1136/ard-2022-223296

[14] Dougados M, Paternotte S, Braun J, Burgos-Vargas R, Maksymowych WP, Sieper J, et al. ASAS recommendations for collecting, analysing and reporting NSAID intake in clinical trials/epidemiological studies in axial spondyloarthritis. Ann Rheum Dis. 2011;70(2):249–51. 20829199 10.1136/ard.2010.133488

[15] Wang R, Bathon JM, Ward MM. Nonsteroidal antiinflammatory drugs as potential disease-modifying medications in Axial Spondyloarthritis. Arthritis Rheumatol. 2020;72(4):518–28. 31705611 10.1002/art.41164 PMC7113090

[16] Consolaro A, Ruperto N, Bazso A, Pistorio A, Magni-Manzoni S, Filocamo G, Malattia C, Viola S, Martini A, Ravelli A. Development and validation of a composite disease activity score for juvenile idiopathic arthritis. Arthritis Care Research: Official J Am Coll Rheumatol. 2009;61(5):658–66. 10.1002/art.24516 19405003

[17] Viola S, Felici E, Magni-Manzoni S, Pistorio A, Buoncompagni A, Ruperto N, et al. Development and validation of a clinical index for assessment of long-term damage in juvenile idiopathic arthritis. Arthritis Rheum. 2005;52(7):2092–102. 15986372 10.1002/art.21119

[18] Weiss PF, Klink AJ, Faerber J, Feudtner C. The pediatric rheumatology quality of life scale: validation of the English version in a US cohort of juvenile idiopathic arthritis. Pediatr Rheumatol. 2013;11(1):43. 10.1186/1546-0096-11-43 PMC3830514 24206654

[19] Pouchot J, Ecosse E, Coste J, Guillemin F, French Quality of Life Study Group, Paediatric Rheumatology International Trials Organisation. Validity of the childhood health assessment questionnaire is independent of age in juvenile idiopathic arthritis. Arthritis Care Res. 2004;51(4):519–26. 10.1002/art.20529 15334422

[20] Indian Academy of Pediatrics Growth Charts Committee, Khadilkar V, Yadav S, Agrawal KK, Tamboli S, Banerjee M, et al. Revised IAP growth charts for height, weight and body mass index for 5- to 18-year-old Indian children. Indian Pediatr. 2015;52(1):47–55. 25638185 10.1007/s13312-015-0566-5

[21] Haq I, Raja M, Ahmad M. A comparison of the 2015 Indian Academy of Pediatrics, International Obesity Task Force and World Health Organization growth references among 5–18-year-old children. Annals Trop Med Public Health. 2017;10:1814.

[22] Marume A, Archary M, Mahomed S. Validation of growth standards and growth references: a review of literature. J Child Health Care. 2022;26(3):498–510. 34114485 10.1177/13674935211024816

[23] Tanner JM. Growth and maturation during adolescence. Nutr Rev. 1981;39(2):43–55. 7010232 10.1111/j.1753-4887.1981.tb06734.x

[24] Morris NM, Udry JR. Validation of a self-administered instrument to assess stage of adolescent development. J Youth Adolesc. 1980;9(3):271–80. 24318082 10.1007/BF02088471

[25] Soliman AT, De Sanctis V. An approach to constitutional delay of growth and puberty. Indian J Endocrinol Metabol. 2012;16(5):698–705. 10.4103/2230-8210.100650 PMC3475892 23087852

[26] Satoh M. Bone age: assessment methods and clinical applications. Clin Pediatr Endocrinol. 2015;24(4):143–52. 26568655 10.1297/cpe.24.143 PMC4628949

[27] Thierry S, Fautrel B, Lemelle I, Guillemin F. Prevalence and incidence of juvenile idiopathic arthritis: a systematic review. Joint Bone Spine. 2014;81(2):112–7. 24210707 10.1016/j.jbspin.2013.09.003

[28] Al-Mayouf SM, Al Mutairi M, Bouayed K, Habjoka S, Hadef D, Lotfy HM, et al. Epidemiology and demographics of juvenile idiopathic arthritis in Africa and Middle East. Pediatr Rheumatol. 2021;19(1):166. 10.1186/s12969-021-00650-x PMC8638433 34857004

[29] Arkachaisri T, Tang SP, Daengsuwan T, Phongsamart G, Vilaiyuk S, Charuvanij S, et al. Paediatric rheumatology clinic population in Southeast Asia: are we different? Rheumatology. 2017;56(3):390–8. 27994096 10.1093/rheumatology/kew446

[30] Tanya M, Teh KL, Das L, Hoh SF, Gao X, Arkachaisri T. Juvenile idiopathic arthritis in Southeast Asia: the Singapore experience over two decades. Clin Rheumatol. 2020;39(11):3455–64. 32418038 10.1007/s10067-020-05081-9

[31] Guzman J, Kerr T, Ward LM, Ma J, Oen K, Rosenberg AM, et al. Growth and weight gain in children with juvenile idiopathic arthritis: results from the ReACCh-Out cohort. Pediatr Rheumatol. 2017;15(1):68. 10.1186/s12969-017-0196-7 PMC5567720 28830457

[32] Jung MK, Song JE, Yang S, Hwang IT, Lee HR. Catch up growth in children born small for gestational age by corrected growth curve. Korean J Pediatr. 2009;52(9):984–90.

[33] Kalra S, Aggarwal S, Kumar A. Counseling for growth hormone therapy. Turkish Archives Pediatr. 2021;56(5):411. 10.5152/TurkArchPediatr.2021.0126 PMC8848749 35110107

[34] Gore AC, Attardi B, DeFranco DB. Glucocorticoid repression of the reproductive axis: effects on GnRH and gonadotropin subunit mRNA levels. Mol Cell Endocrinol. 2006;256(1):40–8. 16839661 10.1016/j.mce.2006.06.002

[35] Whirledge S, Cidlowski JA. A role for glucocorticoids in stress-impaired Reproduction: beyond the Hypothalamus and Pituitary. Endocrinology. 2013;154(12):4450–68. 24064362 10.1210/en.2013-1652 PMC3836069

[36] Tetsuka M, Milne M, Simpson GE, Hillier SG. Expression of 11β-Hydroxysteroid dehydrogenase, Glucocorticoid Receptor, and mineralocorticoid receptor genes in rat Ovary1. Biol Reprod. 1999;60(2):330–5. 9915998 10.1095/biolreprod60.2.330

[37] Ozer Y, Yildiz M, Turan H, Tarcin G, Bingol Aydin D, Gunalp A, et al. Ovarian reserve in children with juvenile idiopathic arthritis using biologic disease-modifying anti-rheumatic drugs. Clin Rheumatol. 2024;43(1):399–406. 37646858 10.1007/s10067-023-06747-w

