# Supplementary material for: Endocrine dysfunction in patients with juvenile idiopathic arthritis
Source: Pediatr Rheumatol Online J. 2025 Apr 24;23:41. doi: 10.1186/s12969-025-01058-7 (PMC12023601; doi:10.1186/s12969-025-01058-7)
Supplement: Supplementary file 1 — Supplementary Material 1 [file 12969_2025_1058_MOESM1_ESM.docx]

**Supplementary material:**

**Section 1: Detailed methodology of laboratory testing**

**Section 2: Tables**

**Table 1:** Multivariate analysis of growth parameters

**Table 2:** Baseline growth parameters (Z scores)

**Table 3:** Factors affecting height, weight & BMI z scores

**Table 4:** Association of categorical variables with anthropometric parameters

**Table 5:** Factors affecting weight & BMI z score

**Table 6:** Multivariate analysis of factors affecting growth parameters (z-score)

**Table 7:** Hormonal assays

**Table 8:** Factors related to age-appropriate Tanner stage attainment

**Table 9:** Associations of categorical variables with age appropriate Tanner stage

**Table 10:** Correlation of height, mid parental height and hormonal SDS

**Section 1:**

**Detailed methodology of laboratory testing:**

After written informed consent from patients as well as from parents, blood samples were freshly collected from the patients in 3 different vials for laboratory testing. 10ml samples were collected from each child and were distributed in EDTA, plain and Fluoride vials for necessary investigations of all patients. Serum Samples were stored in -80℃ for 3-6 months and testing was done for all the samples at a single point of time to nullify percentage of errors related to circumstantial factors like temperature, humidity and buffer pH constitution. Severely haemolysed, turbid and lipemic samples were discarded. Repeated freezing and thawing were avoided. Routine biochemical tests were done on clinical chemistry analyser as per methods given in analyte kit insert. Erythrocyte Sedimentation Rate (ESR) was done in automated ESR analyser. HbA1C (glycosylated haemoglobin) was measured by ion exchange chromatography method (D10 HPLC analyser). C Reactive protein was measured by turbidimetry method. Serum growth hormone, serum IGF-1 (insulin like growth factor -1), IGFBP3 (IGF-1 binding protein 3), anti-Thyroid peroxidase (TPO) antibody, anti-Thyroglobulin (TG) antibody and ACTH levels were investigated by Enzyme Linked Immunosorbent Assay (ELISA) method. Other hormonal assessments like FT4, TSH, Serum cortisol, 25 OH vitamin D, serum Parathyroid hormone, serum fasting insulin, serum FSH, serum LH, serum Testosterone and serum Estradiol were investigated by chemiluminescence microparticle assay method in Automated Immunoassay Analyser. Serum ferritin was also assessed by similar method. Serum lipid profile with Total cholesterol (TC), High Density lipoprotein (HDL), Low density lipoprotein (LDL) and Triglyceride (TG) level estimation was done in the autoanalyzer by Ortho Clinical Diagnostic (OCD) in a NABL accredited laboratory in a dry platform by Precipitation method and the values were estimated by Colorimetric method. Results were analysed with respect to the standardized value for each test. Anti-nuclear antibody (ANA) was tested by indirect immunofluorescence method and reported as pattern and intensity in 1:100 titre. RF (IgM) is checked by immunoturbidimetry method and HLA-B27 by polymerase chain reaction method with the help of specific primer. Serum growth hormone levels were estimated through a solid phase enzyme immunoassay for quantitative determination. XEMA kits were used, and samples were tested according to the package insert instructions and expressed as OD (optical density) values along with the standard and control specimen. Standard curve for growth hormone is depicted below (**Figure 1**).


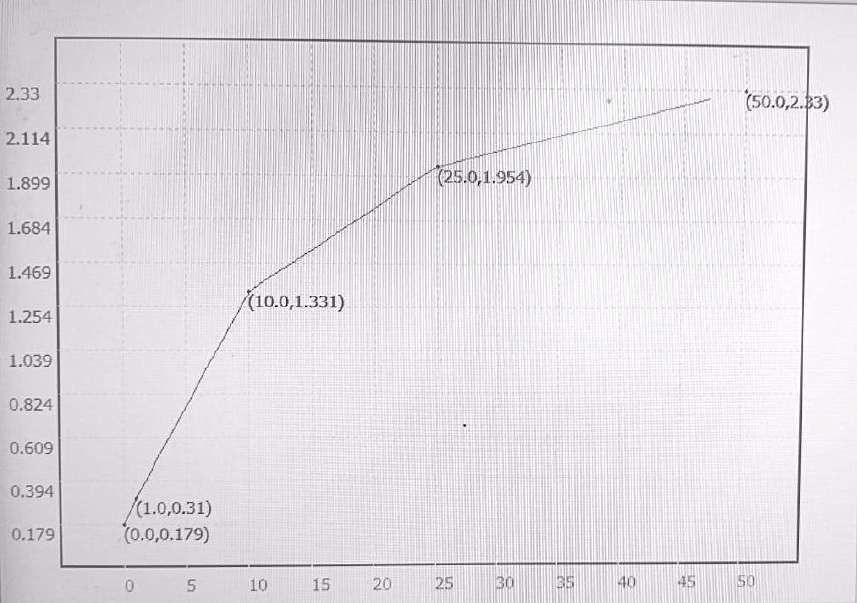


***Figure 1: Standard curve for GH Enzyme linked immunosorbent assay***

Mean and standard deviation for healthy child (1-18 years of age) was 9.9±4.95mIU/l (range 0.2-20mIU/l).

Serum IGF-1 levels were estimated by a separate ELISA kit named DEMEDITEC IGF-1 600 ELISA, which was based on the principle of competitive binding. Patient samples, standards and controls were acidified (50μL0.2MHCl.) and neutralized (10 μL Neutralization Buffer) prior to the assay procedure. The microtiter wells were coated with a monoclonal antibody directed towards an antigenic site on the IGF-1 molecule. During the first incubation, IGF-1 in pre-treated samples compete with an IGF-1-biotin conjugate (100 μL Enzyme Conjugate ) for binding to the coated antibody. After incubation for 120min, the microtiter plate was washed to stop the competition reaction. In the following incubation the bound biotin molecules are detected with streptavidin peroxidase (Enzyme Complex). After a second washing step to remove all unbound substances, the solid phase was incubated with the substrate solution. The colorimetric reaction is stopped by addition of stop solution, and optical density (OD) of the resulting yellow product was measured at 450 nm with a microtiter plate reader.

The intensity of colour was inversely proportional to the concentration of the analyte in the sample. A standard curve was constructed by plotting OD values against concentrations of standards, and concentrations of unknown samples are determined using this standard curve (**Figure 2**). Age and gender wise reference values are also provided (**Figure 3**).

*
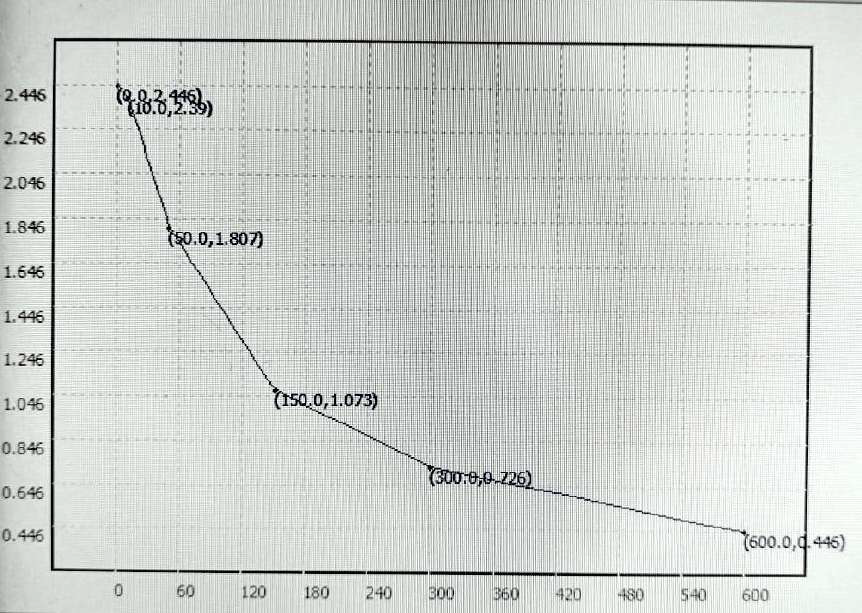
*

***Figure 2: serum IGF-1 standard reference curve based on competitive binding.***

*
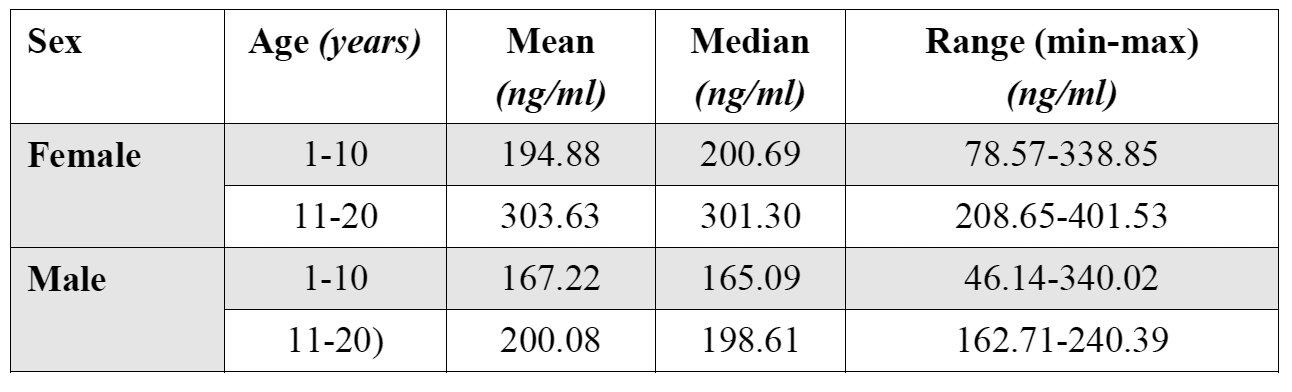
*

***Figure 3: Reference values of human IGF-1 adjusted for age and sex***

Serum IGFBP3 level was measured by ELABSCIENCE sandwich ELISA kit. The test has a sensitivity of 0.47ng/mL and detection range of 0.78-50 ng/mL without significant cross reactivity. Standard curve and reference values adjusted for age are represented in **Figure 4&5**.


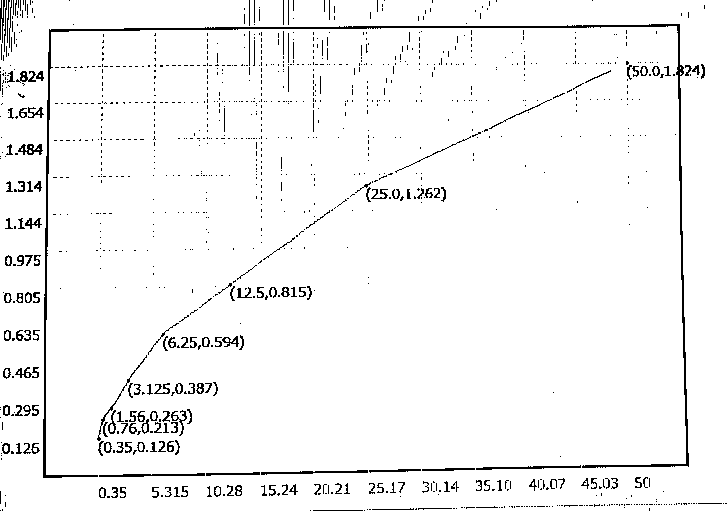


***Figure 4: serum IGFBP3 standard reference curve.***


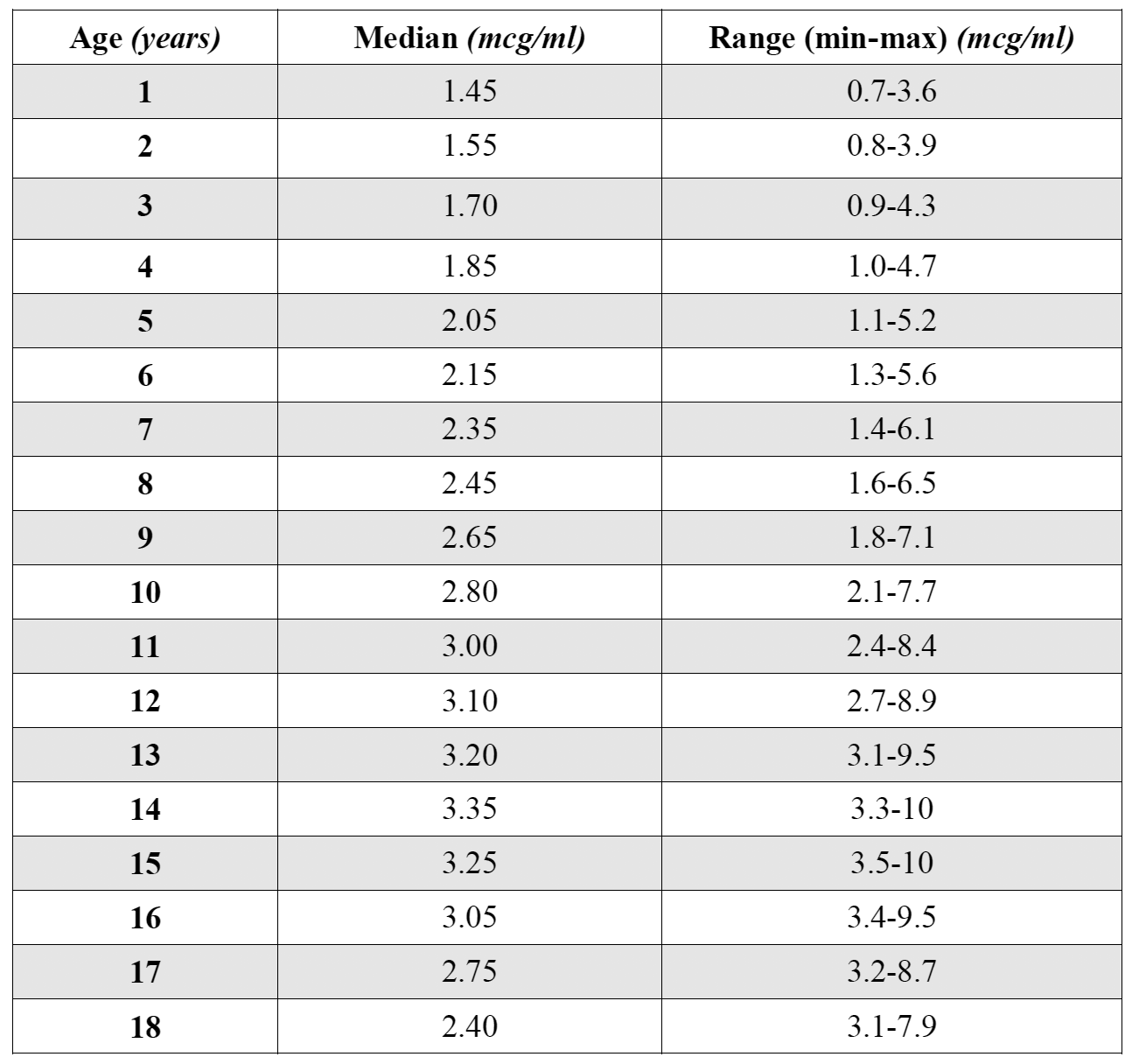


***Figure 5: Reference values of Human IGFBP3 adjusted for age***

Serum GH, serum IGF-1 and IGFBP3 were also expressed as a SDS score which was checked with height SDS score for correlation analysis. SDS scores were formed by applying the following formula for each variable.

***SDS=(X-m)/S***

*Where, X= actual value, m= mean value for adjusted age, sex, S= standard deviation for same adjusted age and sex.*

**Routine blood investigations were carried out in all patients including:**

- Complete blood count
- ESR, CRP
- Fasting blood sugar
- SGOT, SGPT
- Serum creatinine
- Serum calcium & phosphorous
- Serum alkaline phosphatase
- Serum LDH
- Serum Ferritin
- Serum lipid profile
- Serum 25 OH vitamin D
- Urine routine & microscopy

**Mandatory Hormonal profile (for all patients)-**

- Serum GH (growth hormone)
- Serum IGF-1 (insulin like growth factor -1)
- IGFBP3 level (insulin like growth factor binding protein 3)
- Serum TSH (Thyroid stimulating hormone)
- Free T4
- Serum cortisol
- Serum FSH (Follicle-stimulating hormone)
- Serum LH (Luteinizing hormone)
- Serum Testosterone / Serum Estradiol

**Optional laboratory analysis for selected patients –**

- Anti TPO (Thyroperoxidase)
- Anti TG (Thyroglobulin)
- HbA1C
- Serum PTH (Parathyroid hormone)
- Serum ACTH
- Fasting insulin level
- Anti tTG (tissue transglutaminase)

At follow up visit basic investigations were done at 3^rd^, 6^th^, 12^th^ month to observe disease activity and medication side effects as follows: Complete blood count, ESR, CRP, SGOT, SGPT, Serum creatinine.

**Section 2:**

**Tables:**

| **Table 1: Multivariate analysis of growth parameters** | | | | | | |
| --- | --- | --- | --- | --- | --- | --- |
| **Variables** | **Height for age**  **(Short vs. normal)** | | **Weight for age**  **(Underweight vs. normal)** | | **BMI for age**  **(Low vs. normal)** | |
|  | **OR**  **(95% C.I.)** | **p-value** | **OR**  **(95% C.I.)** | **p-value** | **OR**  **(95% C.I.)** | **p-value** |
| Age of onset  (*years*) | 0.951  (0.818-1.106) | 0.52 | 1.087  (0.938-1.260) | 0.27 | 1.019  (0.872-1.191) | 0.81 |
| Duration of illness  (*months*) | 1.000  (0.975-1.025) | 0.98 | 0.974  (0.949-0.999) | **0.04^*^** | 1.023  (0.998-1.049) | 0.08 |
| Current GC dose  (*mg/day*) | 1.027  (0.919-1.148) | 0.64 | 0.983  (0.889-1.087) | 0.74 | 0.979  (0.866-1.108) | 0.74 |
| Cumulative GC dose (*mg*) | 1.000  (0.999-1.001) | 0.49 | 1.000  (0.999-1.001) | 0.70 | 1.000  (0.999-1.001) | 0.60 |
| Duration of GC use (*months*) | 1.063  (0.938-1.205) | 0.34 | 1.018  (0.898-1.154) | 0.78 | 1.051  (0.944-1.169) | 0.37 |
| PGA-VAS | 1.432  (0.803-2.554) | 0.22 | 1.379  (0.776-2.451) | 0.27 | 0.672  (0.389-1.159) | 0.15 |
| PhGA-VAS | 0.651  (0.308-1.378) | 0.26 | 1.299  (0.694-2.431) | 0.41 | 0.780  (0.420-1.448) | 0.43 |
| JADAS27 | 0.986  (0.740-1.313) | 0.92 | 0.751  (0.576-0.978) | **0.03^*^** | 1.301  (1.005-1.683) | **0.04^*^** |
| GH  (*mIU/L*) | 1.029  (0.981-1.079) | 0.25 | 0.986  (0.938-1.037) | 0.58 | 1.001  (0.955-1.050) | 0.96 |
| IGF-1  (*ng/ml*) | 0.996  (0.991-1.001) | 0.12 | 1.002  (0.997-1.006) | 0.52 | 0.999  (0.995-1.004) | 0.78 |
| IGFBP3  (*mcg/ml*) | 0.753  (0.498-1.139) | 0.18 | 1.062  (0.710-1.589) | 0.77 | 0.925  (0.633-1.352) | 0.69 |
| ESR | 1.015  (0.980-1.052) | 0.39 | 1.040  (1.005-1.078) | **0.03^*^** | 0.992  (0.962-1.024) | 0.62 |
| CRP | 0.985  (0.957-1.014) | 0.30 | 0.991  (0.967-1.015) | 0.44 | 0.995  (0.969-1.021) | 0.69 |
| ***p value <0.05 by binary logistic regression model** | | | | | | |

| **Table 2: Baseline growth parameters (Z scores)** | | | | |
| --- | --- | --- | --- | --- |
| **Z scores** | **Categories** | **N (%)** | **Mean (SD)** | **Median**  **(Range)** |
| Height for age z score | | | -1.46 (1.53) | -1.38  (-5.18 to 3.82) |
| Height for age z score | Above -2SD | 75 (70.1) | - | - |
|  | -2SD to -3SD | 14 (13.1) |  |  |
|  | Below -3SD | 18 (16.8) |  |  |
| Weight for age z score | | | -2.04 (1.93) | -1.66  (-10.01 to 1.31) |
| Weight for age z score | Above -2SD | 65 (60.7) | - | - |
|  | -2SD to -3SD | 15 (14.0) |  |  |
|  | Below -3SD | 27 (25.2) |  |  |
| BMI for age z score | | | -1.71 (2.30) | -1.24  (-10.63 to 2.18) |
| BMI for age z score | Above -2SD | 72 (67.3) | **-** | **-** |
|  | -2SD to -3SD | 9 (8.4) |  |  |
|  | Below -3SD | 26 (24.3) |  |  |

| **Table 3: Factors affecting height, weight & BMI z scores** | | | | | | | | | |
| --- | --- | --- | --- | --- | --- | --- | --- | --- | --- |
| **Factors** | **Height z score** | | | **Weight z score** | | | **BMI z score** | | |
|  | **=< -2** | **> -2** | **p-value** | **=< -2** | **> -2** | **p-value** | **=< -2** | **> -2** | **p-value** |
|  | **Mean**  **(SD)** | **Mean**  **(SD)** |  | **Mean**  **(SD)** | **Mean**  **(SD)** |  | **Mean**  **(SD)** | **Mean**  **(SD)** |  |
| GH *(mIU/L)* | 9.56  (9.59) | 7.92  (11.58) | **0.013*** | 8.38  (9.11) | 8.43  (12.14) | 0.176 | 7.70  (9.53) | 8.76  (11.70) | 0.979 |
| IGF-1 *(ng/ml)* | 196.78  (125.95) | 241.1  (140.15) | 0.112 | 208.73  (112.1) | 240.2  (150.46) | 0.339 | 201.07  (110.67) | 240.86  (147.07) | 0.146 |
| IGFBP3 *(mcg/ml)* | 3.32  (1.36) | 3.63  (1.63) | 0.405 | 3.46  (1.55) | 3.59  (1.57) | 0.711 | 3.33  (1.52) | 3.64  (1.57) | 0.351 |
| GH SDS | -0.07  (1.94) | -0.4  (2.34) | **0.013*** | -0.31  (1.84) | -0.3  (2.45) | 0.176 | -0.44  (1.93) | -0.23  (2.36) | 0.979 |
| IGF-1 SDS | -0.19  (4.7) | 1.58  (5.92) | 0.098 | 0.08  (4.16) | 1.68  (6.34) | 0.186 | 0.06  (5.03) | 1.53  (5.86) | 0.075 |
| IGFBP3 SDS | 0.25  (0.81) | 0.6  (1.23) | 0.288 | 0.4  (0.95) | 0.56  (1.24) | 0.781 | 0.32  (0.96) | 0.58  (1.20) | 0.375 |
| JADAS27 | 12.5  (7.5) | 12.6  (8.8) | 0.970 | 13.6  (9.3) | 11.9  (7.8) | 0.497 | 15  (9.60) | 11.40  (7.60) | 0.075 |
| Duration of GC use *(month)* | 5.7  (14.1) | 4.1  (8.4) | 0.625 | 6.2  (12.9) | 3.6  (8.3) | 0.166 | 6.3  (13.60) | 3.80  (8.40) | 0.222 |
| Cumulative GC dose *(mg)* | 653.6  (1766) | 537.4  (983) | 0.545 | 769.5  (1633.7) | 444.7  (938.5) | 0.241 | 765.6  (1667.6) | 478.1  (1006.9) | 0.264 |
| ***p value <0.05 by Mann Whitney U test** | | | | | | | | | |

| **Table 4: Association of categorical variables with anthropometric parameters** | | | | | | | | | | |
| --- | --- | --- | --- | --- | --- | --- | --- | --- | --- | --- |
| **Categories** | | **Stunted** | | | **Underweight** | | | **Low BMI** | | |
|  |  | **No**  **n=85** | **Yes n=22** | **p** | **Yes**  **n=24** | **No**  **n=83** | **p** | **No**  **n=80** | **Yes**  **n=27** | **p** |
|  |  | **N(%)** | **N(%)** |  | **N(%)** | **N(%)** |  | **N(%)** | **N(%)** |  |
| Age of onset (years) | <12 | 45  (52.9) | 18  (81.8) | **0.02*** | 17  (70.8) | 46  (55.4) | 0.18 | 44  (55) | 19  (70.4) | 0.16 |
|  | >=12 | 40  (47.1) | 4  (18.2) |  | 7  (29.2) | 37  (44.6) |  | 36  (45) | 8  (29.6) |  |
| Gender | Male | 60  (73.2) | 12  (54.5) | 0.15 | 16  (66.7) | 56  (67.5) | 0.94 | 52  (65) | 20  (74) | 0.76 |
|  | Female | 25  (26.8) | 10  (45.5) |  | 8  (33.3) | 27  (32.5) |  | 28  (35) | 7  (26) |  |
| Duration of illness  (months) | <24 | 28  (32.9) | 10  (45.4) | 0.27 | 7  (29.2) | 31  (37.3) | 0.46 | 32  (40) | 6  (22.2) | 0.1 |
|  | >=24 | 57  (67.1) | 12  (54.5) |  | 17  (70.8) | 52  (62.7) |  | 48  (60) | 21  (77.8) |  |
| Current GC use | Yes | 22  (25.9) | 9  (40.9) | 0.16 | 10  (41.7) | 21  (25.3) | 0.12 | 22  (27.5) | 9  (33.3) | 0.56 |
|  | No | 63  (74.1) | 13  (59.1) |  | 14  (58.2) | 62  (74.7) |  | 58  (72.5) | 18  (66.7) |  |
| Duration of GC use (months) | <3 | 61  (71.8) | 15  (68.2) | 0.74 | 16  (66.7) | 60  (72.3) | 0.59 | 58  (72.5) | 18  (66.7) | 0.56 |
|  | >=3 | 24  (28.2) | 7  (31.8) |  | 8  (33.3) | 23  (27.7) |  | 22  (27.5) | 9  (33.3) |  |
| Cumulative GC dose (mg) | <1100 | 71  (83.5) | 18  (81.8) | 0.84 | 17  (70.8) | 72  (86.7) | 0.07 | 69  (86.3) | 20  (74.1) | 0.14 |
|  | >=1100 | 14  (16.5) | 4  (18.2) |  | 7  (29.2) | 11  (13.3) |  | 11  (13.7) | 7  (25.9) |  |
| JADAS27 | High +  Moderate | 73  (85.9) | 19  (86.4) | 0.95 | 22  (91.7) | 70  (84.3) | 0.36 | 67  (83.8) | 25  (92.6) | 0.36 |
|  | Low +  inactive | 12  (14.1) | 3  (13.6) |  | 2  (8.3) | 13  (15.7) |  | 13  (16.2) | 2  (7.4) |  |
| JADI-A | <1 | 46  (54.1) | 9  (40.9) | 0.27 | 9  (37.5) | 46  (51.4) | 0.12 | 47  (58.8) | 8  (29.6) | **0.01*** |
|  | >=1 | 39  (45.9) | 13  (59.1) |  | 15  (62.5) | 37  (44.6) |  | 33  (41.2) | 19  (70.4) |  |
| JADI-E | <1 | 58  (68.2) | 12  (54.5) | 0.23 | 10  (41.7) | 60  (72.3) | **0.01*** | 59  (73.8) | 11  (40.7) | **0.01*** |
|  | >=1 | 27  (31.8) | 10  (45.5) |  | 14  (58.3) | 23  (27.7) |  | 21  (26.3) | 16  (59.3) |  |
| ***p<0.05 by chi-square test** | | | | | | | | | | |

| **Table 5: Factors affecting weight & BMI z score** | | | | | | | |
| --- | --- | --- | --- | --- | --- | --- | --- |
| **Factors** | | **Weight z score** | | | **BMI z score** | | |
|  |  | **=< -2** | **>-2** | **p-value** | **=< -2** | **>-2** | **p-value** |
|  |  | **N(%)** | **N(%)** |  | **N(%)** | **N(%)** |  |
| GH | High | 7(16.7) | 3(4.6) | **0.036*** | 8(22.9) | 2(2.8) | **0.001*** |
|  | Normal | 35(83.3) | 62(95.4) |  | 27(77.1) | 70(97.2) |  |
| IGF1 | Low | 10(23.8) | 12(18.5) | 0.504 | 11(31.4) | 11(15.3) | 0.052 |
|  | Normal | 32(76.2) | 53(81.5) |  | 24(68.6) | 61(84.7) |  |
| IGFBP3 | Low | 16(38.1) | 12(18.5) | **0.024*** | 15(42.9) | 13(18.1) | **0.006*** |
|  | Normal | 26(61.9) | 53(81.5) |  | 20(57.1) | 59(81.9) |  |
| Disease  duration | High | 16(38.1) | 22(33.8) | 0.654 | 13(37.1) | 25(34.7) | 0.806 |
|  | Low | 26(61.9) | 43(66.2) |  | 22(62.9) | 47(65.3) |  |
| JADAS27 | High + Moderate | 36(85.7) | 56(86.2) | 0.949 | 31(88.6) | 61(84.7) | 0.591 |
|  | Low + Inactive | 6(14.3) | 9(13.8) |  | 4(11.4) | 11(15.3) |  |
| Duration of GC use (months) | <3 | 28(66.7) | 48(73.8) | 0.424 | 24(68.6) | 52(72.2) | 0.696 |
|  | >=3 | 14(33.3) | 17(26.2) |  | 11(31.4) | 20(27.8) |  |
| Cumulative GC dose (mg) | <1100 | 33(78.6) | 56(86.2) | 0.306 | 27(77.1) | 62(86.1) | 0.245 |
|  | >=1100 | 9(21.4) | 9(13.8) |  | 8(22.9) | 10(13.9) |  |
| ***p value <0.05 by Chi square and Fisher exact t test** | | | | | | | |

| **Table 6: Multivariate analysis of factors affecting growth parameters (z-score)** | | | | | | |
| --- | --- | --- | --- | --- | --- | --- |
| **Variables** | **Height Z-score**  **(<-2SD vs. >-2SD)** | | **Weight Z score**  **(<-2SD vs. >-2SD)** | | **BMI Z score**  **(<-2SD vs. >-2SD)** | |
|  | **OR**  **(95% C.I.)** | **p-value** | **OR**  **(95% C.I.)** | **p-value** | **OR**  **(95% C.I.)** | **p-value** |
| GH  (mIU/L) | 0.976  (0.936-1.018) | 0.26 | 0.997  (0.959-1.036) | 0.87 | 1.005  (0.964-1.048) | 0.82 |
| IGF1  (ng/ml) | 1.002  (0.995-1.009) | 0.64 | 0.999  (0.994-1.005) | 0.65 | 1.001  (0.994-1.008) | 0.80 |
| IGFBP3  (mcg/ml) | 0.404  (0.150-1.090) | 0.07 | 0.597  (0.398-1.361) | 0.22 | 0.940  (0.398-2.223) | 0.89 |
| GH-SDS | 0.887  (0.722-1.091) | 0.26 | 0.984  (0.814-1.190) | 0.87 | 1.024  (0.833-1.259) | 0.82 |
| IGF1-SDS | 1.032  (0.864-1.233) | 0.73 | 1.081  (0.923-1.266) | 0.34 | 1.010  (0.851-1.199) | 0.91 |
| IGFBP3-SDS | 4.434  (1.073-18.319) | **0.04^*^** | 2.205  (0.727-6.688) | 0.16 | 1.392  (0.432-4.489) | 0.58 |
| JADAS27 | 1.008  (0.956-1.064) | 0.76 | 0.975  (0.927-1.025) | 0.32 | 0.944  (0.896-0.995) | **0.03^*^** |
| Duration of GC use  (months) | 0.964  (0.868-1.070) | 0.49 | 0.995  (0.904-1.095) | 0.92 | 0.979  (0.888-1.079) | 0.67 |
| Cumulative GC dose  (mg) | 1.000  (0.999-1.001) | 0.59 | 1.000  (0.999-1.001) | 0.65 | 1.000  (0.999-1.001) | 0.86 |
| Age of onset  (years) | 1.083  (0.937-1.252) | 0.28 | 1.101  (0.968-1.251) | 0.14 | 1.036  (0.910-1.181) | 0.59 |
| ***p value <0.05 by binary logistic regression model** | | | | | | |

| **Table 7: Hormonal assays** *(n=107)* | | | | |
| --- | --- | --- | --- | --- |
| **Tests** | | **N (%)** | **Mean**  **(SD)** | **Median**  **(Range)** |
| Serum GH *(mIU/L)* | | | 8.41 (11.01) | 4.54 (0.14-55.52) |
| Serum GH  *(For age)* | High | 10 (9.3) | - | - |
|  | Normal | 96 (89.7) |  |  |
|  | Low | 1 (0.9) |  |  |
| Serum IGF-1 *(ng/ml)* | | | 227.85 (137) | 201 (13.50-597.90) |
| Serum IGF-1  *(For age)* | High | 29 (27.1) | - | - |
|  | Normal | 56 (52.3) |  |  |
|  | Low | 22 (20.6) |  |  |
| Serum IGFBP3 *(mcg/ml)* | | | 3.54 (1.55) | 3.50 (0.35-8.50) |
| Serum IGFBP3  *(For age)* | High | 1 (0.9) | - | - |
|  | Normal | 78 (72.9) |  |  |
|  | Low | 28 (26.2) |  |  |
| Cortisol *(ug/dl)* | | | 7.70 (4.08) | 7.20 (0.50-20) |
| Cortisol  *(For age)* | Low | 19 (17.8) | - | - |
|  | Normal | 88 (82.2) |  |  |
| Serum FSH *(mIU/L)* | | | 2.91 (2.74) | 1.98 (0.27-16.20) |
| Serum FSH  *(Adjusted for age, sex, and day of cycle)* | High | 3 (2.8) | - | - |
|  | Normal | 88 (82.2) |  |  |
|  | Low | 16 (15.0) |  |  |
| Serum LH *(mIU/L)* | | | 1.75 (1.88) | 1.20 (0.01-9.47) |
| Serum LH  *(Adjusted for age, sex, and day of cycle)* | High | 3 (2.8) | - | - |
|  | Normal | 101 (94.4) |  |  |
|  | Low | 3 (2.8) |  |  |
| Serum Estradiol *(pg/ml)/ (n=35)* | | | 44.74 (41.11) | 29 (3.20-152) |
| Serum Estradiol  *(For age)* | Low | 11 (31.4) | - | - |
|  | Normal | 24 (68.6) |  |  |
| Serum Testosterone *(nmol/L)/ (n=72)* | | | 10.52 (8.78) | 10.65 (0.15-35) |
| Serum Testosterone *(For age)* | Low | 34 (47.2) | - | - |
|  | Normal | 38 (52.8) |  |  |

| **Table 8: Factors related to age-appropriate Tanner stage attainment** | | | | | |
| --- | --- | --- | --- | --- | --- |
| **Factors** | **Age appropriate Tanner Stage** | | | | **p-value** |
|  | **Yes** | | **No** | |  |
|  | **Mean** | **SD** | **Mean** | **SD** |  |
| GH *(mIU/L)* | 7.98 | 11.23 | 9.57 | 10.48 | 0.119 |
| IGF-1 *(ng/ml)* | 230.31 | 149.24 | 221.22 | 98.69 | 0.602 |
| IGFBP3 *(mcg/ml)* | 3.37 | 1.46 | 3.97 | 1.73 | 0.095 |
| GH SDS | -0.39 | 2.27 | -0.07 | 2.12 | 0.119 |
| IGF-1 SDS | 1.49 | 5.94 | -0.11 | 4.53 | 0.132 |
| IGPBP3 SDS | 0.44 | 1.10 | 0.66 | 1.22 | 0.373 |
| JADAS27 | 12.60 | 8.40 | 12.30 | 8.60 | 0.795 |
| GC use *(months)* | 3.90 | 8.00 | 6.60 | 15.10 | 0.586 |
| Cumulative GC dose *(mg)* | 512.70 | 1019.10 | 732.20 | 1767.80 | 0.786 |

| **Table 9: Associations of categorical variables with age appropriate Tanner stage** | | | | | | |
| --- | --- | --- | --- | --- | --- | --- |
| **Variables** | **Categories** | **Age appropriate Tanner Stage** | | | | **p-value** |
|  |  | **Yes** | | **No** | |  |
|  |  | **N** | **%** | **N** | **%** |  |
| GH | High | 7 | 9.0% | 3 | 10.3% | 0.829 |
|  | Normal | 71 | 91.0% | 26 | 89.7% |  |
| IGF1 | Low | 16 | 20.5% | 6 | 20.7% | 0.984 |
|  | Normal | 62 | 79.5% | 23 | 79.3% |  |
| IGFBP3 | Low | 22 | 28.2% | 6 | 20.7% | 0.432 |
|  | Normal | 56 | 71.8% | 23 | 79.3% |  |
| Disease duration | High | 28 | 35.9% | 10 | 34.5% | 0.892 |
|  | Low | 50 | 64.1% | 19 | 65.5% |  |
| JADAS27 | High +  Moderate | 67 | 85.9% | 25 | 86.2% | 0.967 |
|  | Low +  Inactive | 11 | 14.1% | 4 | 13.8% |  |
| Duration of GC use (months) | <3 | 58 | 74.4% | 18 | 62.1% | 0.213 |
|  | >=3 | 20 | 25.6% | 11 | 37.9% |  |
| Cumulative GC dose (mg) | <1100 | 66 | 84.6% | 23 | 79.3% | 0.514 |
|  | >=1100 | 12 | 15.4% | 6 | 20.7% |  |

| **Table 10: Correlation of height, mid parental height and hormonal SDS** | | | | | | |
| --- | --- | --- | --- | --- | --- | --- |
| **Spearman's rho** | | **Height SDS** | **MPH SDS** | **GH SDS** | **IGF-1 SDS** | **IGPBP3 SDS** |
| **Height SDS** | Correlation Coefficient | 1.000 | 0.142 | -0.185 | 0.110 | 0.148 |
|  | **p-value** | - | 0.143 | 0.057 | 0.257 | 0.127 |
|  | N | 107 | 107 | 107 | 107 | 107 |
| **Mid Parental Height (MPH) SDS** | Correlation Coefficient | 0.142 | 1.000 | -0.015 | -0.073 | -0.082 |
|  | **p-value** | 0.143 | - | 0.877 | 0.452 | 0.400 |
|  | N | 107 | 107 | 107 | 107 | 107 |
| **GH SDS** | Correlation Coefficient | -0.185 | -0.015 | 1.000 | 0.008 | .177 |
|  | **p-value** | 0.057 | 0.877 | - | 0.939 | 0.068 |
|  | N | 107 | 107 | 107 | 107 | 107 |
| **IGF-1 SDS** | Correlation Coefficient | 0.110 | -0.073 | 0.008 | 1.000 | 0.174 |
|  | **p-value** | 0.257 | 0.452 | 0.939 | - | 0.072 |
|  | N | 107 | 107 | 107 | 107 | 107 |
| **IGPBP3 SDS** | Correlation Coefficient | 0.148 | -0.082 | 0.177 | 0.174 | 1.000 |
|  | **p-value** | 0.127 | 0.400 | 0.068 | 0.072 | - |
|  | N | 107 | 107 | 107 | 107 | 107 |
