# Supplementary material for: Endocrine dysfunction in patients with juvenile idiopathic arthritis
Source: Pediatr Rheumatol Online J. 2025 Apr 24;23:41. doi: 10.1186/s12969-025-01058-7 (PMC12023601; doi:10.1186/s12969-025-01058-7)
Supplement: Supplementary file 2 — Supplementary Material 2 [file 12969_2025_1058_MOESM2_ESM.docx]

**Response to reviewer's comment**

| **Comment** | **Response** | **Page/para/ line** |
| --- | --- | --- |
| **Reviewer 1** | | |
| '1) when does your practice use steroids for patients? important to explain this (ie more sick patients get steroids? why are children on such long duration of steroid? does everyone who is poly get steroids? sojia?)  This is important because 1) steroids to impact growth 2) steroids can have confounding effect here (ie more sick patients get steroid, therefore do not grow) | - A statement justifying the usage was added in text. - Steroid use is predominantly restricted to SoJIA and some of the erosive Poly JIA, as a bridge therapy but not all. - As you can see there is significant difference between mean and median values of GC use in supplementary table that’s because most of the people was not on any steroid, but SoJIA population received higher dose and that made the average value higher, but Median was 0. - Also, the length of mean use was 2 months, but median was <10 days that’s because of longer use in SoJIA due to unavailability of IL-1 and restricted use of IL-6 in financially unstable patients. - Can’t deny the fact that sicker will get more steroids but we have excluded those who have MAS like features during evaluation. - For evaluating the effect of steroid, we have adjusted values for high vs low dose but haven’t find statistically significant difference. | 5/5/27 |
| 2) I do not think that this work gives insight to growth hormone resistance in JIA necessarily as is written in the results section. GH is elevated in acute and chronic inflammation, regardless of active growing stage or not, GH is in itself an acute phase reactant that increases with metabolic stress, malnourishment, and inflammation. GH regulated immune and inflammatory responses. Making a 1:1 suggestion of failure of growth and GH being elevated isn't quite simple, multiple confounders and pathways between these two in respect to JIA | - Really, it’s a complex situation where you have multiple contributors for a single variable. To neutralize the fact, we have done an analysis of standard deviation score (SDS) for both clinical and hormonal parameter. Correlation analysis showed non-significant poor negative correlation between height and GH SDS score. We have also done multivariate analysis which doesn’t signify any positive indicators. Table added in supplementary file. - Most of the previous literature review includes growth hormone deficiency and treatment with recombinant hormone preparation but we thought its more of resistance at receptor level as GH increased, IGF-1 normal to high and IGFBP3 low. This is just an observation which needs further larger studies for validation of this hypothesis for which our study wasn’t adequately powered. | 6/1/5 |
| 3) what does malnourished mean? patients with increased duration of disease were perhaps more likely to be malnourished- why? how long was first onset of symptom to presentation to rheumatologist? Are those with less access to food less likely to have access to care? we see that in the west all the time | - Term ‘malnourished’ was used for describing low weight for age as in BMI category ‘underweight’ was the classical term used in literature. - Changes have been done to describe low weight for age individuals as ‘underweight’ and low BMI simply expressed as low BMI (<-2SD) as per their age standard. Severity grading was also done for those with below -2SD and -3SD. - As we thought malnourished will not be a proper terminology to express this. - Chronic uncontrolled inflammation may be the driver for poor growth. - Mean disease duration at presentation was 33 months, which showed delay in access to healthcare. Yes, healthcare accessibility is also an issue for the poorer ones. | 6/3/13 |
| 4) you mention coexisting autoimmune diseases such as IBD- these children then do not have JIA but have IBD associated arthritis- the reason they do not grow is far more complicated than it would be in a JIA standpoint. Those with other autoimmunity such as hypothyroidism will have impacted height- and can have elevated GH- this is another confounder | Concomitant diagnosis of other autoimmune conditions was also present in lesser no. of patients. Where the growth dynamics aren’t as simple as JIA. Uncontrolled concomitant endocrinological conditions weren’t included in this study.  The patient of ERA with IBD was diagnosed incidentally as he doesn’t have any GI complaints but screened on basis of family history, high calprotectin value and biopsy evidence of CD. |  |
| **Reviewer 2** | | |
| *Major revisions:* | | |
| Introduction section should be elaborated.  The objective of this study should be clearly explained. | Elaborated and objectives are explained | 2/6/35 |
| Material-methods section should be detailed. | Method section modified and section 1 added in supplementary material expressing for detailing the lab method. |  |
| Results section should be specified.  This section is quite complicated, it should be simplified. This section should be divided to subheadings. | Subheadings are revised and simplified |  |
| Endocrinological analysis should be detailed. It should be explained why other endocrinological evaluations were not performed. | We have checked for the linear growth and pubertal development and markers for those were assessed. Except from that thyroid, cortisol levels were also checked in all patients due to their effect on growth.  We have declared it in the supplementary tables.  Few optional parameters like sr. PTH, sr. ACTH, anti-TPO were also done in specific scenarios but not included in final analysis. | Supplementary table 5. |
| Statistical methods should be checked especially parametric and nonparametric values. Percentage and numerical values should be given consecutively | Numerical and percentages both are presented consecutively in result section. |  |
| Discussion and conclusion section should be specified. Conclusion part of discussion should be added. | Revised |  |
| Diagnostic limitations of this study should be summarized in discussion section. | Limitation of the study is separately mentioned in a para graph. | 10/2/17 |
| *Minor revisions:* | | |
| References section should be updated. References section should be written according to the rules. Authors should cite these references.  ♣    Ozer Y et al. Ovarian reserve in children with juvenile idiopathic arthritis using biologic disease-modifying anti-rheumatic drugs. Clin Rheumatol. 2024 Jan;43(1):399-406. PMID: 37646858.  ♣    Kalra S et al. Counseling for Growth Hormone Therapy. Turk Arch Pediatr. 2021 Sep;56(5):411-414. PMID: 35110107; PMCID: PMC8848749. | Added and reference section updated | Page11 (ref 33 & 37) |
| All abbreviations should be summarized in this manuscript. | List of abbreviations are mentioned | 11/0/0 |
| The keywords should be added by the alphabetical order | Realigned as per alphabetical order | 2/3/5 |
| There are numerous spelling mistakes in the manuscript and reference section. These must be corrected. | Checked and corrected |  |
| Table and figure headings should be more informative. | Headings are revised | Page18-22, supplementary files |
| Manuscript should be reorganized according to journal rules. | Overall reconstruction done according to the journal rules |  |
